# High frequency oscillation network dynamics predict outcome in non-palliative epilepsy surgery

**DOI:** 10.1093/braincomms/fcae032

**Published:** 2024-02-07

**Authors:** Jack Lin, Garnett C Smith, Stephen V Gliske, Michal Zochowski, Kerby Shedden, William C Stacey

**Affiliations:** Neuroscience Graduate Program, University of Michigan, Ann Arbor, MI 48109, USA; Department of Pediatrics, University of Michigan, Ann Arbor, MI 48109, USA; Department of Neurosurgery, University of Nebraska Medical Center, Omaha, NE 68198, USA; Neuroscience Graduate Program, University of Michigan, Ann Arbor, MI 48109, USA; Department of Physics and Biophysics, University of Michigan, Ann Arbor, MI 48109, USA; Department of Statistics and Biostatistics, University of Michigan, Ann Arbor, MI 48109, USA; Department of Neurology, University of Michigan, Ann Arbor, MI 48109, USA; Department of Biomedical Engineering, BioInterfaces Institute, University of Michigan, Ann Arbor, MI 48109, USA; Division of Neurology, Ann Arbor VA Health System, Ann Arbor, MI 48109, USA

**Keywords:** epilepsy, HFO, EEG, network, centrality

## Abstract

High frequency oscillations are a promising biomarker of outcome in intractable epilepsy. Prior high frequency oscillation work focused on counting high frequency oscillations on individual channels, and it is still unclear how to translate those results into clinical care. We show that high frequency oscillations arise as network discharges that have valuable properties as predictive biomarkers. Here, we develop a tool to predict patient outcome before surgical resection is performed, based on only prospective information. In addition to determining high frequency oscillation rate on every channel, we performed a correlational analysis to evaluate the functional connectivity of high frequency oscillations in 28 patients with intracranial electrodes. We found that high frequency oscillations were often not solitary events on a single channel, but part of a local network discharge. Eigenvector and outcloseness centrality were used to rank channel importance within the connectivity network, then used to compare patient outcome by comparison with the seizure onset zone or a proportion within the proposed resected channels (critical resection percentage). Combining the knowledge of each patient’s seizure onset zone resection plan along with our computed high frequency oscillation network centralities and high frequency oscillation rate, we develop a Naïve Bayes model that predicts outcome (positive predictive value: 100%) better than predicting based upon fully resecting the seizure onset zone (positive predictive value: 71%). Surgical margins had a large effect on outcomes: non-palliative patients in whom most of the seizure onset zone was resected (‘definitive surgery’, ≥ 80% resected) had predictable outcomes, whereas palliative surgeries (<80% resected) were not predictable. These results suggest that the addition of network properties of high frequency oscillations is more accurate in predicting patient outcome than seizure onset zone alone in patients with most of the seizure onset zone removed and offer great promise for informing clinical decisions in surgery for refractory epilepsy.

## Teaser

Lin *et al*. report that network properties of epileptic high frequency oscillations are effective at predicting seizure freedom when compared with the proposed clinical resection margin in patients with definitive epilepsy surgery.

## Graphical Abstract

**Graphical Abstract:**
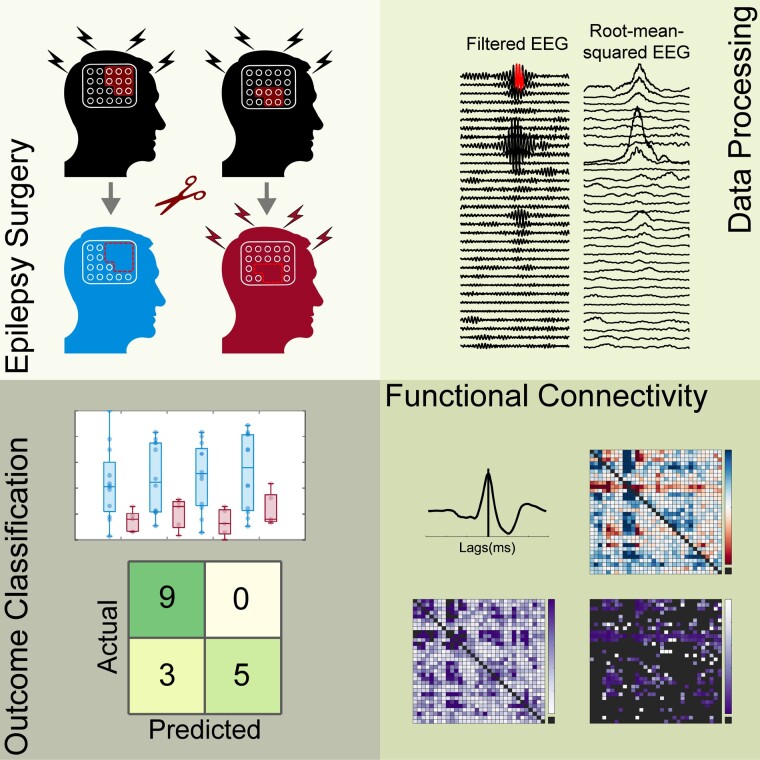

## Introduction

Every year, tens of millions of people around the world suffer from epilepsy.^1,2^ This common neurological disorder affects people of all ages and is characterized by chronic seizures that are debilitating and deadly to some individuals. While advances in pharmacotherapy in the past few decades have improved side effects and tolerability, roughly one-third of the patients remain unresponsive to any kind of drug treatment.^3,4^ In patients with intractable epilepsy, the standard of care is to determine whether epilepsy surgery is possible. Ideally, the surgery removes the offending brain tissue and seizures would resolve. However, only 35–80% of these patients achieve seizure freedom after surgery, depending on the type of surgical resection.^5-7^ The cause of these surgical failures is often unclear. Suboptimal outcomes are expected when the surgery is merely palliative, i.e. the clinicians used their clinical judgment to identify the seizure onset zone (SOZ), but then could not resect all of it for various reasons.^8^ However, even when all of the SOZ is resected, there are still many patients who continue to have seizures. In other words, the clinical standard of care—the SOZ—is not an ideal prognostic biomarker of surgical outcome.^9^ This has led to the search for additional prognostic biomarkers of outcome.^10,11^ Two promising biomarkers are high frequency oscillations (HFOs)^12-14^ and network connectivity.^15-17^ Here, we combine these two approaches, evaluating the network properties of HFOs to develop a useful biomarker of outcome in epilepsy surgery.

HFOs are 80–500 Hz waveforms that last less than 100 ms.^12,18^ HFOs were first discovered in healthy rodent hippocampus and later shown to also be prevalent in humans.^19,20^ Many later studies showed that HFOs are elevated within the SOZ in patients with epilepsy^18,21-23^ and that resecting channels with high HFO rate was associated with good surgical outcome.^14,21,24^ It has been challenging to translate these results into clinical practice. One intriguing method is to use HFOs to predict the outcome of epilepsy surgery. This method was the foundation of the first prospective clinical study using HFOs, which calculated the rate of all channels during 10 min of slow wave sleep. Unfortunately, that information was unable to predict patient outcome, even when restricting the analysis to the higher frequency ‘fast ripples’ that had been proposed as being more specific.^25^ Later work by the same group found that fast ripples showed promise to predict surgical outcome if the analysis was expanded to several hours of data.^26^ Another clinical trial compared using HFO rate with spike rate to tailor surgical margins, but found that HFOs were not superior to spikes.^27^ Thus, despite a wealth of data showing correlation of HFO rate with outcome, it is still unclear how to use these data clinically.

All of those prior studies had an important limitation: they analysed HFOs on each channel independently. Yet epilepsy is a network disorder wherein the electrographic signals propagate across channels.^28-30^ Clinicians avoid reading EEGs as single channels because they know that it is critical to see the interactions between different channels. We hypothesize the HFOs have similar network interactions. A few prior studies have shown that HFOs exhibit propagation, with the earliest HFO onset channels being more correlated with the SOZ, but they did not quantify the network properties.^31,32^ Several groups analysed the functional connectivity of background ictal and interictal EEG in the context of HFO location or frequency bands, but did not analyse the connectivity of the HFOs themselves.^33-35^ Another recent study showed that two functional connectivity metrics of individual fast ripples (200–600 Hz HFOs) could improve the rate of misclassifying surgical outcome prediction by at least 50%.^36^ These results suggest that incorporating network information will improve the efficacy of using HFOs as a predictive biomarker of surgical outcome.

We first characterized the HFO networks using functional connectivity analysis, which is used to understand the connections between different regions of the brain.^37-40^ Subsequently, we analysed these networks using a set of graph theory algorithms commonly used in various disciplines including social, transportation or biological networks for identifying important nodes, known as centralities.^41-43^ We aimed to see how well channel centrality of interictal HFOs could predict surgical outcome. We performed two different comparisons. The first was to measure centrality within the SOZ, similar to how previous researchers considered HFO rates. This helps determine if interictal HFO data, which comprises the vast majority of the EEG recording, can capture similar information to the SOZ, which requires waiting for seizures to occur. However, as described above, SOZ is an imperfect biomarker of outcome—our goal is to provide novel information, not simply identify the SOZ channels that the clinicians are already searching for. In order to fit this tool into the clinical workflow, we designed a new measurement that compares the overlap of the HFO data with the tissue that is proposed to be resected, which we named critical resection percentage (CReP). These two measurements test how well all information available to a clinician prior to resection [HFOs, SOZ, proposed resected volume (RV)] can be used to predict surgical outcome. Our results show that this method is readily translatable to clinical workflows and predicts outcomes better than both SOZ and HFO rates.

## Materials and methods

### Patient population

**Figure 1:**
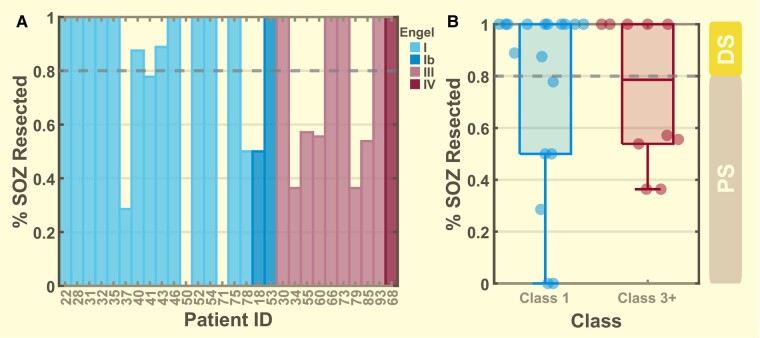
**Distribution of patient seizure onset zone resection percentage.** (**A**) Individual patient SOZ resection percentage coloured by their Engel outcome classification. (**B**) Patients divided into two groups with Class 1 (*n* = 18) made up of Engel I and Ib and Class 3+ (*n* = 10) made up of Engel III and IV. For Class 1, 12 of the 18 patients had at least 80% SOZ resection and for Class 3+, 5 of the 10 patients had at least 80% SOZ resection. Patients with at least 80% SOZ resection are grouped into the DS category while those less than 80% are placed into the PS category.

All consecutive patients from the University of Michigan who had undergone intracranial EEG implantation for refractory epilepsy monitoring between 2016 and 2022 were evaluated (*n* = 68). Only a rare minority of epilepsy surgeries are performed without receiving intracranial EEG (e.g. obvious right mesial temporal sclerosis). Inclusion criteria were (1) eventual resective surgery and (2) the reference standard of Engel outcome classification of class I, III or IV after 1 year.^44^ Patients with good (I) outcome were grouped into Class-1 while those with poor outcome (III, IV) were grouped into Class-3+. As Class-2 patients (*n* = 7) are characterized as having ambivalent outcome and have been included as either ‘good’ and ‘bad’ outcomes in past work,^14,22,45,46^ we have excluded them from most of the analyses. Patients receiving neurostimulation devices (*n* = 27) were excluded, as were patients who did not have surgical resection (*n* = 6). This resulted in a total of 28 available Class-1 (*n* = 18) and Class-3+ (*n* = 10) patients for our analyses (Fig. 1). We further divided the patients into two groups based on how much of their SOZ was resected [non-palliative definitive surgery (DS): 80%, palliative surgery (PS): < 80%, see next section]. This results in 17 DS (12 Class-1, 5 Class-3+) and 11 PS (6 Class-1, 5 Class-3) patients. Of the seven Class-2 patients, six were DS. All patient data used in this study are approved by the local Institutional Review Board with written patient consent to share and use their de-identified information.

### Seizure onset zone resection percentage

Across all 28 patients, there were 18 good outcomes (class I) and 10 poor outcomes (nine class III, one class IV). After determining which electrodes were resected, we noted that many of the surgeries left a large portion of the SOZ behind. Throughout this paper, the SOZ is defined as the channels in which the seizures begin, as listed in the official clinical report and vetted by consensus of at least eight epilepsy physicians in the conference. The SOZ is used directly by the treating clinicians to determine where to perform the surgical resection in each patient. While the goal of the surgery is to attempt to remove all the SOZ, sometimes a portion of these channels cannot be resected due to clinical scenarios such as anatomic constraints and eloquent cortex. In such patients, the clinicians anticipate and accept a PS with lower chance of seizure freedom. It is difficult to assess outcome in such patients. In contrast, when most or all of the SOZ is resected, the clinical team expects a good outcome. Therefore, we separated the patients into DS and PS groups with DS being represented by patients with 80% of SOZ resected and PS by those with 80% of the SOZ resected. The 80% threshold was chosen in a data-driven manner: 10/12 of the DS patients had 100% SOZ resection, the other two had 87.5% and 90%, and changing the threshold between 80% and 100% did not affect results. Our analyses focused primarily on the DS patients: here, the clinicians believe the patient has the best chance for success based on all available data.

### Data acquisition

All data are from intracranial electrodes. Implanted electrodes consisted of a mixture of stereo-EEG depth, subdural grids and conventional depth electrodes that were chosen based on standard clinical care for each patient (Supplementary Table 1). All channels were monitored at a sampling rate of 4096 Hz with a Quantum amplifier (Natus Medical Inc.) with signals referenced to the lab standard placed between the Fz and Cz. Clinically-defined SOZ, surgical outcomes and seizure times were obtained through clinical reports and consultation with the treating clinicians. Only data > 30 min outside of seizure times were used in this study. Determination of which channels were included in the RV was performed using Curry (Compumedics) software and coregistering the post-implant and post-resection imaging; two trained epilepsy physicians (WS, GS) adjudicated when electrode volumes were > 50% removed by the resection. The final determination of each patient’s SOZ and RV was reached through the consensus of at least three epileptologists, who presented the case to a conference of at least five other epileptologists at the presiding hospital.

### Automated HFO detection

All HFO detections were obtained using a previously-validated detection system.^47-49^ Very briefly, the HFO detections were performed using the automated, root-mean-square-based *Staba* detector^50^ with improved specificity for ‘true’ (neural tissue generated) HFO by redacting detections overlapping with periods of sharp transients, widespread events that are most likely due to noise and other signal-based artefacts.^49^ We used a common average reference, including all grid or depth electrodes as a single group, as described in Gliske *et al*. 2016.^49^ Additionally, we ignored HFO detections that are coincident with muscle-based EMG artefacts using another validated detector.^47^ These algorithms were all previously validated by showing a high level of agreement with trained human reviewers for distinguishing between true HFOs and artefactual HFOs.^49^ These multiple levels of artefact rejection were necessary to allow inclusion of HFO data from all interictal times and brain states.

We include HFOs from all brain states, which is facilitated by our algorithm that removes artefacts.^47,49^ We note that artefactual HFOs would be expected to introduce noise to our analysis and reduce the effect size, so the strong correlations with surgical outcome present herein suggest these methods are successful at identifying HFOs correlated with epileptic tissue.^51^

### Ranked HFO rate

Most prior HFO work has focused on rates on individual channels. We used that measurement (HFO-RATE) as a benchmark for our network measurements, to show whether these new network data add information to prior HFO work. To quantify the HFO rate, we counted the number of interictal HFOs detected on every channel over the course of the patient’s stay at the hospital and performed an ordinal ranking where the lowest HFO count channel is ranked as zero with the highest HFO count channel ranked up to *N*−1 and then normalized by the largest rank so the normalized ranked score ranged from zero to one. This totalled over 11 million HFOs detected (Table 1). Further details on the following methods are found in Supplementary Materials. Supplementary Fig. 1 summarizes the entire process.

**Table 1:** Patient demographics and clinical data

Patient	Age	Sex	ILAE class	Seizure focus (hemisphere, region)	Pathology	Percent SOZ resection	DS	Total HFO count
**UMHS-0018**	41	M	1	L F	CD	50%	N	110 177
**UMHS-0019**	59	F	2	R T	Gliosis	100%	Y	124 642
**UMHS-0020**	45	F	3	R T	MTS	100%	Y	28 734
**UMHS-0022**	40	M	1	L T	CD, MTS	100%	Y	63 700
**UMHS-0025**	17	F	2	L T	Gliosis	80%	Y	178 705
**UMHS-0028**	14	F	1	R T	Tumour: Glioma	100%	Y	216 041
**UMHS-0030**	5	M	5	L T	MTS, Gliosis	100%	Y	441 168
**UMHS-0031**	13	M	1	L T	Gliosis, Tumour: NF1	100%	Y	569 313
**UMHS-0032**	41	F	1	R F	CD	100%	Y	542 990
**UMHS-0033**	5	F	4	R Insula	CD, Gliosis	100%	Y	86 855
**UMHS-0034**	33	F	5	R F	Gliosis	36%	N	421 877
**UMHS-0035**	50	F	1	L Hipp.	Gliosis	100%	Y	127 121
**UMHS-0037**	14	M	1	L F	DNET	29%	N	227 536
**UMHS-0038**	28	M	2	L T	MTS, Gliosis	100%	Y	215 510
**UMHS-0040**	14	F	1	L *P*	CD, Gliosis	87.5%	Y	377 528
**UMHS-0041**	32	F	1	R F	CD	78%	N	86 499
**UMHS-0042**	17	M	4	L Insula		37.5%	N	31 465
**UMHS-0043**	28	M	1	R T	Gliosis	89%	Y	493 238
**UMHS-0046**	23	F	1	L F	CD	100%	Y	14 762
**UMHS-0047**	48	F	2	R T	Gliosis	100%	Y	236 590
**UMHS-0050**	31	F	1	L Hipp.	Gliosis	0%	N	287 992
**UMHS-0052**	27	M	1	L Hipp.	MTS, Gliosis	100%	Y	164 878
**UMHS-0053**	55	F	1	R T	Gliosis	100%	Y	481 267
**UMHS-0054**	35	F	1	L Hipp.	Gliosis	100%	Y	171 316
**UMHS-0055**	42	M	5	L T, Hipp.	HS, Gliosis	57%	N	753 699
**UMHS-0060**	23	M	5	R T	Tumour: RG, Gliosis	56%	N	1 090 979
**UMHS-0066**	43	F	5	R F	FCD, Gliosis	100%	Y	1 011 536
**UMHS-0068**	23	F	6	R Hipp.		100%	Y	650 594
**UMHS-0071**	56	F	1	L Hipp.	Gliosis	0%	N	290 862
**UMHS-0073**	39	F	5	L Hipp.	Gliosis	100%	Y	194 040
**UMHS-0075**	24	M	1	L T	Gliosis, MTS	100%	Y	802 164
**UMHS-0078**	35	M	1	L Hipp.	MTS	50%	N	337 362
**UMHS-0079**	25	F	5	L Hipp.		36%	N	887 655
**UMHS-0085**	21	M	5	R F	FCD	54%	N	648 016
**UMHS-0093**	8	M	5	R *P*	FCD	100%	Y	101 452
					**Totals**			12 468 273
					**Averages**			356 236.1

CD, cortical dysplasia; DNET, dysembryoplastic neuroepithelial tumour; F, frontal; FCD, Frontal cortical dysplasia; Hipp., Hippocampus; HS, hippocampal sclerosis; L/R, left/right; M/F, male, female; MTS, medial temporal sclerosis; NF1, neurofibromatosis type 1 tumour; P, parietal; PMG, polymicrogyria; PVNH, periventricular nodular heterotopia; RG, recurrent ganglioglioma; RV, Resected volume (# electrodes within); T, temporal.

### Functional connectivity analysis

**Figure 2:**
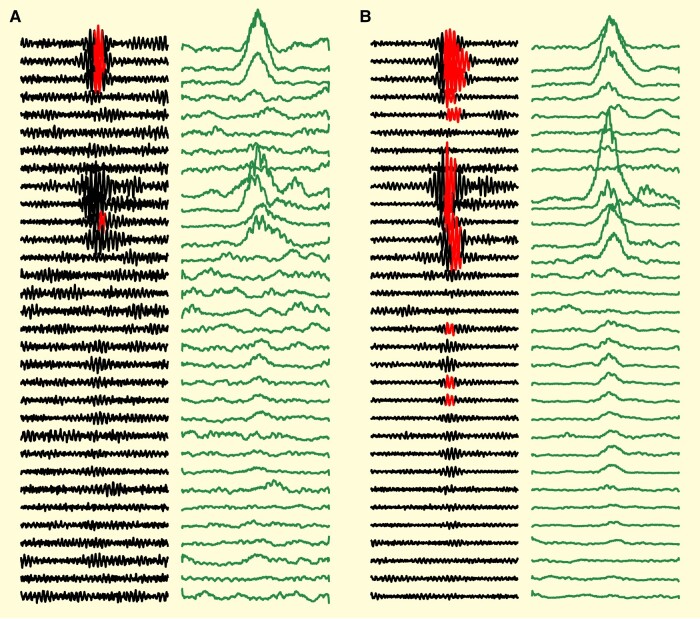
**Data analysis examples.** Filtred (80–500 Hz) EEG (black) with superimposed HFO detections (red overlay) show how neighbouring channels often had high frequency discharges that did not cross threshold for the HFO detector but were still part of the network spread. In order to remove the effect of oscillatory phase and standardize the time of onset, cross-correlation analysis was performed on the root-mean-squared filtered data (green panels to right). (**A**) ‘Sample’ with a network discharge that had detected HFOs in three channels, but likely involved at least seven. Note that even in detected channels, the red detection does not accurately detect the onset/offset time of the true discharge. (**B**) Sample showing network HFO discharge over several channels simultaneously with varying amplitudes.

To perform the network analysis, we randomly selected 500 HFOs from each channel independently. As shown in Fig. 2, many HFOs arose within network discharges across multiple channels, and several channels that were clearly involved in the discharge did not have ‘detected’ HFOs. In addition, the start and end times of the HFOs were often not an accurate representation of the entire discharge time, which would make calculating lag between channels unreliable. Therefore, we converted the analysis to use the actual EEG data itself. To this end, each sample of data is taken as an N*-*channel by 100 ms array of EEG data centred around the time at which an HFO was detected. Each channel was first re-referenced against the common average reference of their respective channel type (grid or depth electrodes) to remove spurious large amplitude, global artefacts like head scratching or movement. We then applied a 60 Hz IIR comb filter and an 80–500 Hz elliptical passband filter. Finally, we calculated the root mean squared of the data with 10 ms sliding windows to further obtain the magnitude of the high frequency activity (Fig. 2).

Using these 500 ‘samples’ from each channel, we first generated a functional connectivity network (FCN) that characterized the mean of peak correlation amplitudes between each electrode pair, normalized to the background. Since individual HFOs are produced by small volumes of brain tissue, we assumed any zero-lag correlation across channels to be due to volume conduction or artefact, rather than propagating HFOs.^52-54^ Thus, we ignored all correlations at lags between −1 ms and 1 ms. We then generated a lag asymmetry network (LAN) that measured how likely each channel’s HFOs were to lead or lag the activity in other channels. The LAN distributions were estimated with a kernel density estimation with a 95% pointwise confidence interval via bootstrapping.^55,56^ To account for the requirements of the centrality measures, the LAN was further divided into an undirected network (uLAN) that measured the peak connection between each electrode pair and a forward network (fLAN) that only measured forward propagation (negative lag). All values from all three networks (FCN, uLAN, fLAN) were normalized by converting them to ordinal ranks (0:*N*−1 channels), and dividing the score by the highest rank. For more detail on the methodology, please consult the Supplementary material.

### Centrality

Centrality is a category of tools used in network theory to evaluate the role of each node within a network.^57-59^ Eigenvector centrality was performed in both networks (FCN-EIG and uLAN-EIG), which measured the importance or influence of each channel within the network.^45-47,60^ Outcloseness centrality was performed in the fLAN network (fLAN-OUT) to measure how far ‘upstream’ each channel is compared to the others.^43,57,58^

### HFO features

The HFO-RATE, FCN-EIG, uLAN-EIG and fLAN-OUT comprise four ‘HFO features.’ For each feature, all channels within each subject were ordinally ranked from 0 to *N*−1 based on the raw value, then the rank was divided by the highest-ranking channel. Each channel then has a single rank for each of the four features.

### Clinical prediction algorithm

#### SOZ feature score

We evaluated how well the four measurements (HFO-RATE, FCN-EIG, uLAN-EIG and fLAN-OUT) could distinguish between good outcome (Class-1) and poor outcome (Class-3+) patients. We accomplished this by computing the mean rank for each feature in (1) every channel in the SOZ (-SOZALL); (2) the highest-ranking 50% of channels within the SOZ (-SOZ50); (3) the highest ranking 10% of channels within the SOZ (-SOZ10); or (4) the single highest ranked channel in the SOZ (-SOZTOP). This produced a four-dimensional feature vector for each subject, for each group of features.

#### Critical resection percentage

To evaluate how well the centralities and HFO rate relate with resected volume, we calculated how many of the highest ranked (i.e. most important) channels would be resected. The strategy is designed to fit within the clinical decision protocol: identify which channels are proposed to be removed, then compare if that resection would include the ‘important’ channels using the features above.

**Figure 3:**
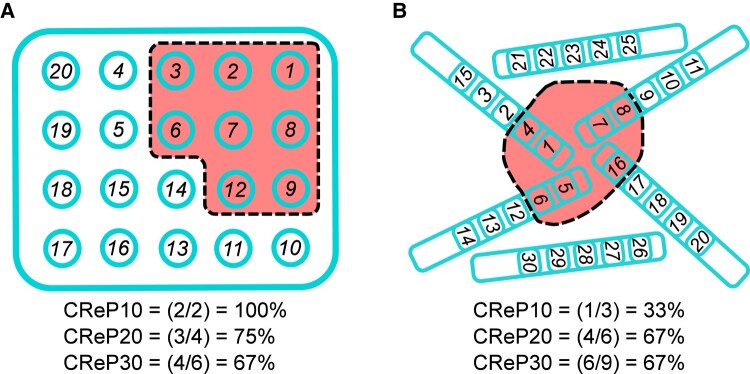
**Critical resection participation calculation examples.** (**A**) Given a grid layout of 20 electrodes (teal circles) in which eight electrode positions will be resected (red region), we first rank each electrode based upon importance (e.g. highest HFO rate or highest centrality). The ranks are shown as numbers on each electrode. We then determine how many of the top *N*% (e.g. 10%, 20%, 30%) of those ranks will be resected. The CReP10 results in two of the top 2 (10% of 20 electrodes = 2, so CReP10 is ‘what percent of the top 2 ranks is resected’). Since ranks 1 and 2 are both resected, CReP10 = 100%. For CReP20, it is out of four electrodes (20*20%), and since ranks 1, 2 and 3 are resected but not 4, the CReP20 is 3/4 = 75%. CReP30 is four of the top six (20*30%). (**B**) A similar example with depth electrodes. CReP10 results in one of the top three (30*10%) ranked electrodes being resected, CReP20 with four of the top six (30*20%) electrodes being resected, and CReP30 with six of the top nine (30*30%) electrodes being resected.

This CRePX (where *X* = 10, 20, 30 or 40) is the fraction of a specified *X*% of the highest ranked channels that are within the proposed resection margins, i.e. ‘how many of most important *X*% channels will be resected?’ To calculate CReP, you need to know the total number of electrodes, the specific electrodes that are going to be resected, and for each HFO measurement, the rank of every channel in the planned resection. For a given percentage *X*, the number of channels to evaluate *n_CReP_* = ceiling (N_total channels_ * *X*%). CRePX = (number of channels in resected volume with rank n_CReP_th highest rank)/*n_CReP_*. Note that rank hierarchy is in reverse numerical order, i.e. rank 1 is the ‘highest’. Also note that the maximum value of CRePX is limited by the number of resected channels, which may be less than *n_CReP_*, i.e. the maximum CREP20 when 10 channels out of 87 channels are resected is 10 (resected)/18 (i.e. 20% of 87 channels) = 0.556. See Fig. 3 and Supplementary material for examples. We evaluated four different percentiles of channels: the top 40% (-CReP40), 30% (-CReP30), 20% (-CReP20) and 10% (-CReP10). These were performed for each of the HFO measurements (FCN-EIG, uLAN-EIG, fLAN-OUT, HFO-RATE).

#### Classification with Naïve Bayes model

Using the Engel classifications as response variables, we combined the different measures into a single algorithm designed to predict, as the margins for surgery are being planned, whether the patient is likely to have Class 1 versus Class 3+ outcome. It is important to note that all values used in this algorithm (SOZ, CReP, HFO measures) are known prior to the planned surgery. To build a classifier with multiple features, we opted for Naïve Bayes as it has the advantage of assessing the contribution of each feature in the model and provides a probability score for the prediction belonging to a given class. Specific features were chosen based upon the results of the Hotelling’s T in the prior sections. A leave-one-out Naïve Bayes was performed (each patient left out for testing once) using the selected features and the resulting posterior probabilities were used to construct a receiver operating characteristic (ROC) curve to assess the model’s discrimination performance on each left-out patient. Calibration of the model was then assessed by plotting the predicted posterior probability against the observed probability of having a Class 1 outcome. To test our model in a real-world, prospective-like manner, held-out patients in the form of the Class 2 DS (*n* = 6) patients were treated as separate test inputs against each of the leave-one-out models.

### Statistical analysis

To evaluate the differences in a given set of stratified SOZ or CReP measurements between good and poor surgical outcome patients, we employed Hotelling’s T2 used for multivariate hypothesis testing (= 0.05). In the cases where there is a significant difference in the omnibus test, *post hoc* analysis for each individual feature was subsequently done with two-tailed Student’s *t*-test (=0.05). Additionally, we measured the effect size for distinguishing Class 1 versus Class 3+ across the whole patient cohort using the area under the ROC curve (AUC) wherein a random classifier (i.e. distinguishing two classes that have the same distribution) would have an AUC = 0.5. For the prediction of surgical outcome, we employed a Naïve Bayes model (see above section for more information) with leave-one-out cross-validation in which the posterior probabilities were used to construct a ROC curve for evaluating the performance of the classifier. The significance of the AUC of the ROC was evaluated through bootstrap hypothesis testing against the null hypothesis of AUC = 0.5, which denotes a random classifier, and the confidence interval was computed using the Hanley–McNeil method.

## Results

### HFO centrality and rate in SOZ compared with patient outcome

**Figure 4:**
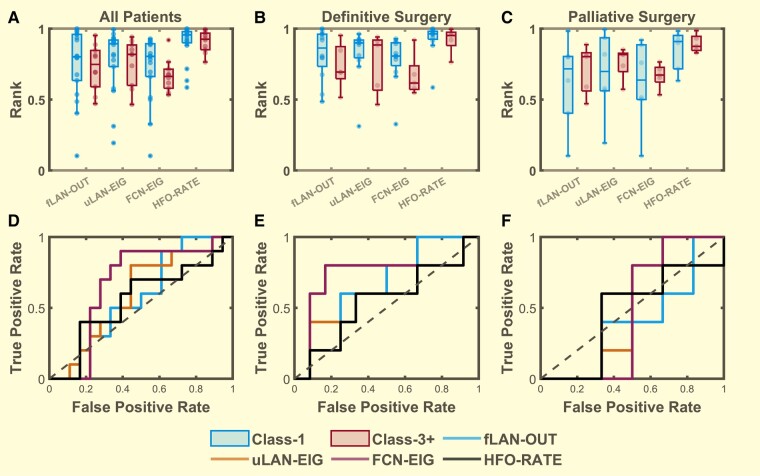
**Distribution of the top 50% highest seizure onset zone (SOZ50) mean centrality ranks.** Comparison between Class 1 (blue, *n* = 18) and Class 3+ (red, *n* = 10) for (**A**) all patients, (**B**) definitive surgery patients (*n* = 12,5), (**C**) palliative surgery patients (*n* = 6,5) with SOZ50 computed for the outcloseness centrality (fLAN-OUT) of the forward propagating network, eigenvector centrality (uLAN-EIG) of the undirected lag asymmetry network, eigenvector centrality (FCN-EIG) of the FCN, and the ranked HFO rate (HFO-RATE). While it seems that the mean SOZ centrality ranks and ranked HFO rates (HFO-RATE) are generally higher in class-1 definitive surgery group compared to that of the class-3+ group there was no statistical significance detected for group differences for each stratified patient sets (all patients, definitive surgery and palliative surgery) (*Hotelling’s T2 P* = 0.914, 0.227, 0.966; *T2* = 0.975, 5.652, 0.572; *n* = 28, 17, 11, respectively). (**D–F**) The ROC curves were used to demonstrate the effect size for each feature in distinguishing between class-1 and class-3+ for each respective stratified patient sets (**A–C**). Boxes represent the interquartile range with the horizontal line being the median and whiskers extending 1.5 times the interquartile range. Jittered data points are overlaid on top of the box plots.

We first tested relationships in the entire cohort of 28 patients. Many studies have shown that channels with high HFO rate channels are in the clinically-defined SOZ. Here, we wanted to see if HFO propagation network features also corresponded with the SOZ, with the added goal of being able to distinguish between good (Class-1) and bad outcome (Class-3+) patients. Each of the functional connectivity graphs (FCN, uLAN, fLAN) representing relational information between each electrode pair generates unique centrality values at each channel (FCN-EIG, uLAN-EIG, fLAN-OUT). Together with the HFO-RATE, this makes four HFO measures that characterize each channel. For each of these measures, we calculated the mean centrality rank of the top 50% most important SOZ channels (SOZ50), resulting in four features. Although we saw that there was not a significant group difference between the Class-1 and Class-3+ patients (*P* > 0.05, *n = 28*), we noted that overall, the Class-1 centrality and HFO-RATE scores tended to be slightly higher than those of Class-3+ (Fig. 4A and D). This was repeated for the mean of all SOZ scores (SOZALL), the mean of the top 10% highest SOZ scores (SOZ10) and the single highest SOZ scores (SOZTOP) with all showing relatively similar results but no significant group difference (*P > 0*.05, *n = 28*) (Supplementary Fig. 2).

### HFO centrality and rate in critical resection percentage compared with patient outcome

**Figure 5:**
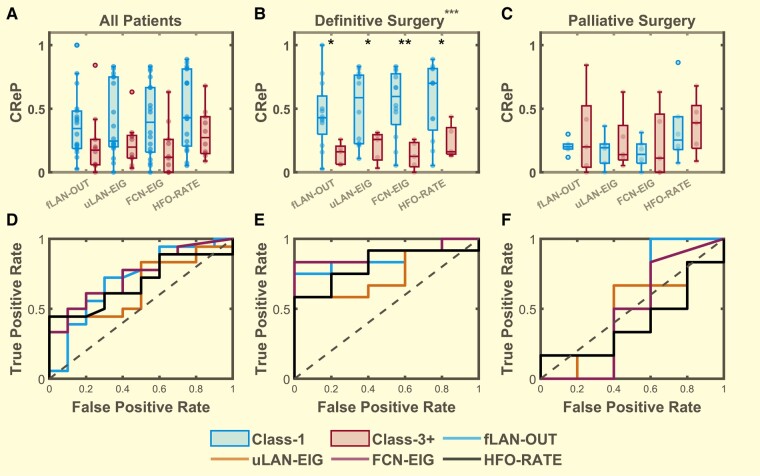
**Distribution of the overlap of the resected volume with the top 30% (CReP30) highest-ranked channels.** CReP calculated for the outcloseness centrality (fLAN-OUT) of the forward propagating network, eigenvector centrality (uLAN-EIG) of the uLAN, eigenvector centrality (FCN-EIG) of the FCN, and the ranked HFO rate (HFO-RATE) comparing between Class 1 (blue, left) and Class 3+ (red, right) for (**A**) all patients (Class 1, 3 *n* = 1810), (**B**) definitive surgery patients (*n* = 12,5), (**C**) palliative surgery patients (*n* = 6,5). Statistically significant main effects of group differences were detected using Hotelling’s T2 for the definitive surgery group (*P* = 1.25 * 10^−5^*, T2* = 27.996*, n* = 17) but not for the all patients group (*P* = 0.136, *T2* = 7.002, *n* = 28) nor the palliative surgery group (*P* = 0.978, *T2 =* 0.454, *n* = 11)*. Post hoc* comparisons using *t*-test were performed for each individual features within the definitive surgery group as the main effect was found to be significant (*P <* 0.05, *t >* 2.2, *n* = 17 *for all features*). Significant differences were denoted by * for *P <* 0.05, ** for *P <* 0.01 and *** for *P <* 0.001. Boxes represent the interquartile range with the horizontal line being the median and whiskers extending 1.5 times the interquartile range. Jittered data points are overlaid on top of the box plots. (**D**–**F**) ROC curves computed to show the effect sizes for each individual features for comparing between Class-1 and Class-3+ for each patient set above (**A**–**C**). (**E**) The AUC of the definitive surgery group ROC curves = 0.85 (95% CI = 0.663, 1.037, *n = 17*), 0.75 (95% CI = 0.507, 0.993, *n* = 17), 0.9 (95% CI = 0.75, 1.05, *n* = 17) and 0.817 (95% CI = 0.609, 1.024, *n* = 11), respectively for fLAN-OUT, uLAN-EIG, FCN-EIG and HFO-RATE.

Given the limitations of comparing SOZ with outcome, we developed a method to compare directly with the planned resection. We compared each of our HFO measures with RV to see how well they could distinguish between Class 1 and Class 3+ patients by calculating the CReP in the 28 patients. Specifically, we calculated the CReP from the top 30% most important channels (CReP30) ranked by centrality and HFO-RATE scores respectively. By comparing these features between Class-1 and Class-3+ patients, we saw that patients with good outcome generally have a higher CReP30 than that of patients with poor outcome (Fig. 5A and D). While this difference between the groups was determined to not be significant, we noted it to have a moderate trend (*P =* 0.1358, *n = 28*). Likewise, this was repeated for CReP calculated from the top 40% (CReP40), top 20% (CReP20) and top 10% (CReP10) most important channels with trends of group differences detected at CReP20 (*P* = 0.103, *n* = 28) and CReP10 (*P* = 0.061, *n = 28*) (Supplementary Fig. 2).

### SOZ network centrality scores in patients with definitive surgery

As shown in Fig. 1B, there was a disparity in the amount of SOZ resected in different patients, leading to a natural stratification. We focused on the DS patients because they were expected to become seizure free (see Discussion). Once again for each of the centrality (FCN-EIG, uLAN-EIG, fLAN-OUT) and HFO-RATE measures, we computed the mean rank of the top 50% most important SOZ channels (SOZ50) to derive four features. Here we saw a more prominent differentiation of the SOZ features for outcome with Engel Class 1 patients (DS-1) generally having higher scores than that of class 3 or 4 patients (DS-3+), although the difference was yet again not significant (*P* > 0.05, *n* = 17) (Fig. 4B and E). These were repeated for SOZALL, SOZ10 and SOZTOP with all showing no significant group differences (*P* > 0.05, *n* = 17) (Supplementary Fig. 3). This suggests that while there is some enrichment of the good outcome features relative to the poor outcome features, comparison with SOZ is not a strong predictor of surgical outcome. For completeness, the same analysis was also performed for the PS patients which resulted in a significant group difference within the SOZTOP (*P* = 0.029, *n* = 11) feature set although *post hoc t*-test revealed no significant differences for any features (*P* > 0.05, *n* = 11) (Fig. 4C and F and Supplementary Fig. 4). We thus conclude that comparing HFO centrality and rate with SOZ is not a reliable predictive biomarker of surgical outcome.

### High critical resection percentage in definitive surgery patients

We computed the CReP from the top 30% most important channels (CReP30) for each of our four measures (FCN-EIG, uLAN-EIG, fLAN-OUT, HFO-RATE) for the DS patients. DS-1 patients generally had higher CReP than that of DS-3+ (Fig. 5B and E). A global Hotelling’s test showed a strongly significant difference between the two patient classes (*P <* 0.0001, *n* = 17) with *post hoc t*-test showing significant differences between the outcomes for every feature (*P <* 0.05, *n* = 17). These analyses were repeated for the other percentile groups (CReP 40, CReP 20 and CReP10) with each showing significant group differences (*P <* 0.05, *n =* 17) (Supplementary Fig. 3). These calculations were also performed in the PS cohort; no significant differences were detected between the classes (*P* > 0.05, *n =* 11) (Fig. 5C and F and Supplementary Fig. 4).

### Patient classification with network centrality features

**Figure 6:**
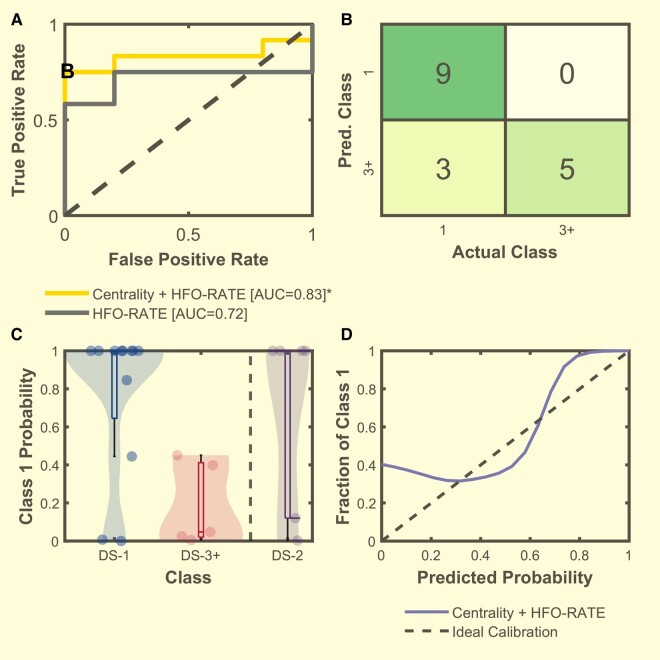
**Naïve Bayes model results for the classification of patient outcome for DS patients.** (**A**) The ROC curves computed from the leave-one-out Naïve Bayes validation set posterior probabilities for the model with the CReP30 feature based on HFO-RATE (grey) and the model with CReP30 of centralities and HFO-RATE (yellow). The statistical significance of the AUC of the ROC curve was evaluated for both curves. While the AUC of the HFO-RATE ROC was not significantly different (AUC = 0.72, 95% CI = 0.459, 0.974, *P* = 0.138 via bootstrap test, *n =* 17), the AUC of the centrality + HFO-RATE ROC was significantly different from a random classifier (AUC = 0.83, 95% CI = 0.636, 1.031, *P* = 0.008 via bootstrap testing, *n* = 17). (**B**) Confusion matrix computed from the chosen point ‘B’ in A with perfect specificity and an accuracy of 83%. (**C**) Violin plot of the leave-one-out Naïve Bayes posterior probabilities for the model with centralities features and HFO-RATE. Boxes represent the interquartile range with the horizontal line being the median and whiskers extending 1.5 times the interquartile range. Jittered data points are overlaid on top of the box plots. Results for DS-1 (*n* = 12) and DS-3+ (*n* = 5) patients are from leave-out cross-validation. DS-2 patient data are the mean and standard deviations of each patient (*n* = 6) to all 17 cross-validation models (see Supplementary Fig. 6). (**D**) Calibration curve of the centrality + HFO-RATE model. This plot demonstrates that the algorithm is very accurate when it is more certain (higher probability) of the outcome.

These results demonstrated that features based on the RV may be good predictors of surgical outcome for definitive surgery patients. To test this, we built a classifier to distinguish DS-1 from DS-3+ patients. We selected the set of CReP30 features as they demonstrated a very significant global difference between surgical outcomes. We then trained a Naïve Bayes model with a combination of the CReP30 for the four features (fLAN-OUT, uLAN-EIG, FCN-EIG, HFO-RATE), and performed leave-one-out cross-validation on each of the 17 patients. The AUC of the cross-validated model was 0.83 (Fig. 6A), which was significantly better than a random classifier with an AUC of 0.5 (*P =* 0.008 bootstrap hypothesis test, 95% CI = 0.636, 1.031, *n* = 17). This ROC curve shows excellent specificity: at threshold ‘B’ there are no false positives, i.e. all nine predictions of good outcome were correct (see confusion matrix in 4B, corresponding to the point B in 4A). Additionally, the algorithm showed a high positive predictive value (PPV) of 100% (9/9) at the 80% predicted class 1 certainty (Fig. 6C) which significantly (*P* = 0.007, permutation test) outperforms prediction of surgical outcome based on clinical data of SOZ resection percentage with a PPV of 71% (12/17) at 80% SOZ resection. However, as the certainty drops, the algorithm becomes less accurate. This is assessed via a calibration curve (Fig. 6D). For comparison, to see how well HFO-RATE performs alone we built a Naïve Bayes model with just CReP30 HFO-RATE with leave-one-out cross-validation as we did above and obtained an AUC of 0.72, which was not significantly different from a random classifier with an AUC of 0.5 (*P* > 0.05 bootstrap test, 95% CI = 0.459, 0.974, *n* = 17). Additionally, the PPV at the 80% predicted class 1 certainty for HFO-RATE-only classifier was 88% (7/8) and was not significantly different than predicting outcome based on SOZ resection percentage (*P =* 0.122, permutation test) (Supplementary Fig. 5). This suggests that HFO-RATE alone is not greatly predictive of surgical outcome. Overall, these results show that a classifier based on a combination of HFO centrality and rate features can be used to determine post-surgical outcomes with a high level of specificity for definitive surgery patients.

**Figure 7:**
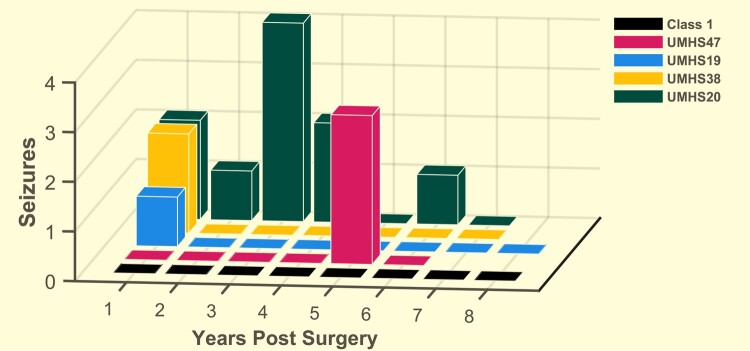
**Number of seizures per year post-surgery for all patients predicted to have good outcome.** Combining the results of the Naïve Bayes algorithm in Fig. 6, there were 13 patients predicted to have good outcome. Nine of them were Class 1 and did not have any seizures after surgery for 6–8 years (black) (*n* = 9). The other four were Class 2, three of whom had seizure-free periods more than 4 years long (UMHS47, 19, 38).

Until this point, we have excluded Class 2 patient data from all analyses because it is ambiguous whether to include them as ‘good’ or ‘bad’ outcome, and thus it is unclear how to utilize those data when building a classifier. However, once the classifier is created, it is important to assess how it would work with all patients including Class 2. We therefore applied the Naïve Bayes classifiers to all DS Class 2 patients (*n* = 6). Due to the cross-validation, there were 17 classifier models. We averaged the response of each DS-2 patient to all models to show their predicted probability (Fig. 6C). There was minimal variability across the model predictions (Supplementary Fig. 6). We found that 4/6 of the DS-2 patients were predicted to be class 1 with mean 100% probability, while the other two were predicted to have poor outcome. Examining all six patients in aggregate, we compared the CReP30 features across DS-1, DS-2 and DS-3+ (Supplementary Fig. 7). Class 2 patients were significantly different than Class 3+ patients (*n* = 11, *P* < 0.05 Hotelling’s T2), but not than Class 1 patients (*n* = 17, *P* = 0.0181, *P* > 0.05 Hoteling’s T2). We also assessed the clinical response of each of these six patients individually to see if there was a difference between those with predicted good and bad outcomes (Fig. 7). We found that of the four with predicted good outcome, three of them had prolonged seizure-free periods (>4 years) and only had a single, brief period of breakthrough seizures. We were conservative and included these patients as Class 2 despite the prolonged seizure freedom. The fourth has had 10 seizures in 7 years (previously had > 30 seizures per year).

## Discussion

In this article, we evaluated HFO network propagation properties as a predictive biomarker of surgical outcome. Our results demonstrate that detection of HFO functional networks is complementary with HFO rate to predict patient outcome within definitive surgery patients. The high accuracy of these measures allowed us to seek an even more important goal: to see if incorporating HFO data, which are invisible to clinicians and not part of the standard of care,^61^ could predict outcome better than current clinical methods. We used several objective HFO measurements to build a cross-validated Naïve Bayes model that successfully predicts patient outcome based upon the planned resection margins in patients where most of the SOZ were resected.

One of the previous possible limitations of HFOs’ effectiveness as a clinical tool may be that prior research has been restricted to HFO rate on single channels. Epilepsy is recognized as a network disorder, in which the interactions between channels is critical to evaluating the epileptic network.^28,30,62^ Many studies have shown that different centrality measures can quantify epileptic networks within standard EEG to be potential biomarkers for epileptic tissue.^40,45,46,63,64^ Similar analyses in the context of HFOs are also promising, as analysing the connectivity of EEG in the HFO frequencies or HFOs themselves shows correlation with patient outcome or can improve specificity to epilepsy.^31-36^ However, network analysis with HFOs is challenging because some channels have few events, and most of the detected events on one channel are not detected on neighbouring channels even when there is a clear network discharge—which is precisely the data that are most important to analyse the network (e.g. Fig. 2). In the current work, we used the unique approach of characterizing functional HFO networks using our automated HFO detections as a marker for time windows to perform correlational analysis of all high frequency intracranial EEG activity across all channels.

This work presents several novel findings that can improve future work with HFOs and other EEG biomarkers. The first is stratification of patients into definitive versus palliative surgery. The primary purpose of implanting intracranial EEG is to determine the SOZ and decide what to resect. The overlap between those two decisions, although crucial, is rarely reported. In our cohort, which we assume is similar to most centres, there are two very different groups of patients: those in whom the SOZ was resected (DS) and those where it was not (PS). As shown in Fig. 1B, 55% of our PS patients (6/11) had good outcomes, which may actually be better than clinicians were hoping. This is compared with 71% (12/17) good outcomes in the DS patients, which is probably worse than clinicians were hoping but is typical for surgical programs.^65^ It is notable that five of the Class 3+ patients had 100% of their SOZ resected, and two Class 1 patients had none of it resected. This is a critical clinical scenario: the outcome in these patients was unrelated to the SOZ resection. While the good outcome in a PS patient is a welcome surprise, the failure in the DS patients is frustrating for clinicians and patients. It would be highly useful to identify poor outcomes before the surgery. This scenario is an ideal testbed to analyse the potential role of HFOs: can HFO data help distinguish which patients will have good or bad outcomes in patients with complete resection of the SOZ? In other words, can HFOs add information to standard clinical practice, beyond what is already available to the treating physicians? We propose that new biomarkers of surgical outcome should focus on the DS group, where the ‘standard of care’ (resecting the full SOZ) is being met, to determine if the new biomarkers might improve upon that standard. Here, we note that because the decision of where to resect is integrally connected to the clinically-defined SOZ, it is impossible to predict if a patient would remain as DS or PS if a different number of SOZ channels had been chosen. However, a review of each patient’s electrode numbers showed the assignation to DS/PS would most likely remain unchanged even with random changes in the SOZ channels (data not shown).

The second contribution is to demonstrate that comparing HFOs—or any potential biomarker—with SOZ is not an ideal method for predicting outcome. Most prior HFO work has looked for correlations with the SOZ, which is readily available and easily testable, but is highly dependent on reviewer^61^ and is often not a fair representation of the surgical resection. Although numerous studies have shown that interictal HFO rates are higher in SOZ versus non-SOZ tissue, and that resection of these areas corresponds to good surgical outcome,^18,21-23^ the first prospective study was unable to predict which patients would have good outcome using this method.^25^ Concordantly, our results also suggest that while HFO-based SOZ features show some minor trends that correlate with outcome groups, these trends are not significant enough to identify which patients will have good outcome. Furthermore, from a translational point of view, one other concern is that simple comparison with SOZ merely tries to recapitulate the standard of care, rather than providing new complementary information. In short, the clinical team is already finding the SOZ, and it has known limitations in predicting outcome—it is not an ideal benchmark for a biomarker meant to improve care.

Those concerns about SOZ led us to develop an additional measurement that is *independent and complementary to the SOZ*. Our CReP measurement compares the percent of overlap between a biomarker and the proposed resection. Not only is the SOZ sometimes not fully resected, but the surgical resection usually removes electrodes outside the SOZ, which affects outcome independent of the SOZ. However, past work that compared with the entire resected volume suffered from low specificity because many ‘unimportant’ channels are also resected (e.g. removing the entire right temporal lobe when only hippocampal electrodes are abnormal).^49,66^ Thus, the CReP focuses on a percentile of the most important channels. We find that CReP features are better at distinguishing between surgical outcomes than the SOZ features and are promising as a predictor of good outcome.

This method is designed to provide clinicians with a probability of surgical success during the most critical time of surgical planning, after all information has been acquired and they decide what to resect. Our tool is designed to be a ‘final check’ that might identify epileptic networks that were not visible using standard tools. As a pseudo-prospective way of testing the performance of our classifier, we tested our model against Class 2 patients who were initially held out of the model due to their inconsistent grouping from other studies.^14,22,45,46^ We showed that Class 2 patients that we classified with a high probability of being a good outcome patient coincide with the low post-surgical seizure resurgences further validating our model. We cannot directly combine the cross-validation (Class 1, Class 3+) and held-out data (Class 2). However, when viewed together we estimate that a pseudo-prospective analysis of all 23 DS patients (Classes 1, 2, 3+) would have predicted a good outcome in 13 patients (nine Class 1, four Class 2), and 12 of those would have been seizure free for at least 4 years.

However, we do recognize some limitations here including the existence of a moderate class imbalance with more DS-1 patients than DS-3+ patients due to our strict criteria. Another limitation, which is present in all HFO research, is that it is still unclear how to identify ‘normal’ versus ‘abnormal’ HFOs.^67^ Regardless, our resulting classifier was able to achieve high accuracy in our cohort while maintaining a high level of specificity. Finally, although our analysis could not test this, a straightforward next step will be to determine if altering the planned resection to include key channels can improve outcome. This is readily testable within the model and can be implemented in future clinical trials.

In summary, we offer a different perspective for translating HFOs as a biomarker for epilepsy by characterizing their interictal HFO functional networks. We identified that restricting analysis to patients with definitive surgery is a better demonstration of how HFOs can predict outcome. Within this clinical context of definitive surgery, our results indicate that spatial–temporal patterning of HFOs forms functional networks, and that the addition of network centrality was more predictive of outcome than HFO rate alone. SOZ was not a reliable way to predict outcome, so we developed a novel tool, CReP, which was very successful. These findings together have broad implications to the development of HFOs, as well as other EEG signals, as predictive biomarkers of surgical outcome. Our findings further bolster the status of HFO as a promising biomarker for epilepsy and provide a basis for how HFO can be used as a clinical tool informing clinical decisions.

## Supplementary material

Supplementary material is available at *Brain Communications* online.

## Supplementary Material

fcae032_Supplementary_Data

## Data Availability

Code to process the data with detailed descriptions and full pipeline available at: https://github.com/J4KLin/HFO-Network.git. Data for two patients available at the University of Michigan Deep Blue Data: https://doi.org/10.7302/n9rt-sc45.

## References

[1] Fiest KM, Sauro KM, Wiebe S, et al Prevalence and incidence of epilepsy. Neurology. 2017;88(3):296–303. 27986877 10.1212/WNL.0000000000003509 PMC5272794

[2] Beghi E, Giussani G, Abd-Allah F, et al Global, regional, and national burden of epilepsy, 1990–2016: A systematic analysis for the global burden of disease study 2016. Lancet Neurol. 2019;18(4):357–375. 30773428 10.1016/S1474-4422(18)30454-X PMC6416168

[3] Kwan P, Brodie MJ. Early identification of refractory epilepsy. New Engl J Med. 2000;342(5):314–319. 10660394 10.1056/NEJM200002033420503

[4] Laxer KD, Trinka E, Hirsch LJ, et al The consequences of refractory epilepsy and its treatment. Epilepsy Behav. 2014;37:59–70. 24980390 10.1016/j.yebeh.2014.05.031

[5] Mohan M, Keller S, Nicolson A, et al The long-term outcomes of epilepsy surgery. PLoS One. 2018;13(5):e0196274. 29768433 10.1371/journal.pone.0196274 PMC5955551

[6] Wiebe S, Blume WT, Girvin JP, Eliasziw MA. Randomized, controlled trial of surgery for temporal-lobe epilepsy. New Engl J Med. 2001;345(5):311–318. 11484687 10.1056/NEJM200108023450501

[7] Widjaja E, Jain P, Demoe L, Guttmann A, Tomlinson G, Sander B. Seizure outcome of pediatric epilepsy surgery: Systematic review and meta-analyses. Neurology. 2020;94(7):311–321. 31996452 10.1212/WNL.0000000000008966

[8] Huang C, Marsh ED, Ziskind DM, et al Leaving tissue associated with infrequent intracranial EEG seizure onsets is compatible with post-operative seizure freedom. J Pediatr Epilepsy. 2012;1(4):211–219. 24563805 10.3233/PEP-12033 PMC3930198

[9] Gliske S V., Stacey WC. The BEST conceivable way to talk about epilepsy biomarkers. Epilepsy Curr. 2023;23:175–178. Ahead of Print. 37334422 10.1177/15357597231159714 PMC10273820

[10] 2014 NINDS Benchmarks for Epilepsy Research. National Institute of Neurological Disorders and Stroke. Published July 25, 2022. Accessed 4 October 2022. https://www.ninds.nih.gov/about-ninds/strategic-plans-evaluations/strategic-plans/2014-ninds-benchmarks-epilepsy-research

[11] Rummel C, Abela E, Andrzejak RG, et al Resected brain tissue, seizure onset zone and quantitative EEG measures: Towards prediction of post-surgical seizure control. PLoS One. 2015;10(10):e0141023. 26513359 10.1371/journal.pone.0141023 PMC4626164

[12] Bragin A, Engel J, Wilson CL, Fried I, Mathern GW. Hippocampal and entorhinal cortex high-frequency oscillations (100–500 Hz) in human epileptic brain and in kainic acid-treated rats with chronic seizures. Epilepsia. 1999;40(2):127–137. 9952257 10.1111/j.1528-1157.1999.tb02065.x

[13] Worrell GA, Parish L, Cranstoun SD, Jonas R, Baltuch G, Litt B. High-frequency oscillations and seizure generation in neocortical epilepsy. Brain. 2004;127(7):1496–1506. 15155522 10.1093/brain/awh149

[14] Jacobs J, Staba R, Asano E, et al High-frequency oscillations (HFOs) in clinical epilepsy. Prog Neurobiol. 2012;98(3):302–315. 22480752 10.1016/j.pneurobio.2012.03.001 PMC3674884

[15] Terry JR, Benjamin O, Richardson MP. Seizure generation: The role of nodes and networks. Epilepsia. 2012;53(9):e166-9. 22709380 10.1111/j.1528-1167.2012.03560.x

[16] Stacey W, Kramer M, Gunnarsdottir K, et al Emerging roles of network analysis for epilepsy. Epilepsy Res. 2020;159:106255. 31855828 10.1016/j.eplepsyres.2019.106255 PMC6990460

[17] Ramantani G, Westover B, Gliske S, et al Passive and active markers of cortical excitability in epilepsy. Epilepsia. 2023;64 Suppl 3:S25–S36. 36897228 10.1111/epi.17578 PMC10512778

[18] Jacobs J, Zelmann R, Jirsch J, Chander R, Dubeau CÉCF, Gotman J. High frequency oscillations (80–500 Hz) in the preictal period in patients with focal seizures. Epilepsia. 2009;50(7):1780–1792. 19400871 10.1111/j.1528-1167.2009.02067.x PMC3764053

[19] Frauscher B, von Ellenrieder N, Zelmann R, et al High-Frequency oscillations in the normal human brain. Ann Neurol. 2018;84(3):374–385. 30051505 10.1002/ana.25304

[20] Ylinen A, Bragin A, Nádasdy Z, et al Sharp wave-associated high-frequency oscillation (200 hz) in the intact hippocampus: Network and intracellular mechanisms. J Neurosci. 1995;15(1 I):30–46. 7823136 10.1523/JNEUROSCI.15-01-00030.1995 PMC6578299

[21] Haegelen C, Perucca P, Châtillon CE, et al High-frequency oscillations, extent of surgical resection, and surgical outcome in drug-resistant focal epilepsy. Epilepsia. 2013;54(5):848–857. 23294353 10.1111/epi.12075 PMC3712982

[22] Cho JR, Koo DL, Joo EY, et al Resection of individually identified high-rate high-frequency oscillations region is associated with favorable outcome in neocortical epilepsy. Epilepsia. 2014;55(11):1872–1883. 25266626 10.1111/epi.12808

[23] Liu S, Gurses C, Sha Z, et al Stereotyped high-frequency oscillations discriminate seizure onset zones and critical functional cortex in focal epilepsy. Brain. 2018;141(3):713–730. 29394328 10.1093/brain/awx374 PMC6715109

[24] Höller Y, Kutil R, Klaffenböck L, et al High-frequency oscillations in epilepsy and surgical outcome. A meta-analysis. Front Hum Neurosci. 2015;9:574. 26539097 10.3389/fnhum.2015.00574 PMC4611152

[25] Jacobs J, Wu JY, Perucca P, et al Removing high-frequency oscillations: A prospective multicenter study on seizure outcome. Neurology. 2018;91(11):e1040–e1052. 30120133 10.1212/WNL.0000000000006158 PMC6140372

[26] Nevalainen P, Von Ellenrieder N, Klimeš P, Dubeau F, Frauscher B, Gotman J. Association of fast ripples on intracranial EEG and outcomes after epilepsy surgery. Neurology. 2020;95(16):e2235–e2245. 32753439 10.1212/WNL.0000000000010468 PMC7713787

[27] van ‘t Klooster MA, Leijten FSS, Huiskamp G, et al High frequency oscillations in the intra-operative ECoG to guide epilepsy surgery (“the HFO trial”): Study protocol for a randomized controlled trial. Trials. 2015;16(1):422. 26399310 10.1186/s13063-015-0932-6 PMC4581519

[28] Kramer MA, Cash SS. Epilepsy as a disorder of cortical network organization. Neuroscientist. 2012;18(4):360–372. 22235060 10.1177/1073858411422754 PMC3736575

[29] Stam CJ . Modern network science of neurological disorders. Nat Rev Neurosci. 2014;15(10):683–695. 25186238 10.1038/nrn3801

[30] Scharfman HE, Kanner AM, Friedman A, et al Epilepsy as a network disorder (2): What can we learn from other network disorders such as dementia and schizophrenia, and what are the implications for translational research? Epilepsy Behav. 2018;78:302–312. 29097123 10.1016/j.yebeh.2017.09.016 PMC5756681

[31] Tamilia E, Park EH, Percivati S, et al Surgical resection of ripple onset predicts outcome in pediatric epilepsy. Ann Neurol. 2018;84(3):331–346. 30022519 10.1002/ana.25295

[32] González Otárula KA, von Ellenrieder N, Cuello-Oderiz C, Dubeau F, Gotman J. High-frequency oscillation networks and surgical outcome in adult focal epilepsy. Ann Neurol. 2019;85(4):485–494. 30786048 10.1002/ana.25442

[33] Ibrahim GM, Anderson R, Akiyama T, et al Neocortical pathological high-frequency oscillations are associated with frequency-dependent alterations in functional network topology. J Neurophysiol. 2013;110(10):2475–2483. 24004529 10.1152/jn.00034.2013

[34] Zweiphenning WJEM, van ‘t Klooster MA, van Diessen E, et al High frequency oscillations and high frequency functional network characteristics in the intraoperative electrocorticogram in epilepsy. Neuroimage Clin. 2016;12:928–939. 27882298 10.1016/j.nicl.2016.09.014 PMC5114532

[35] Rijal S, Corona L, Perry MS, et al Functional connectivity discriminates epileptogenic states and predicts surgical outcome in children with drug resistant epilepsy. Sci Rep. 2023;13(1):9622. 37316544 10.1038/s41598-023-36551-0 PMC10267141

[36] Weiss SA, Fried I, Wu C, et al Graph theoretical measures of fast ripple networks improve the accuracy of post-operative seizure outcome prediction. Sci Rep. 2023;13(1):367. 36611059 10.1038/s41598-022-27248-x PMC9825369

[37] Bullmore E, Sporns O. Complex brain networks: Graph theoretical analysis of structural and functional systems. Nat Rev Neurosci. 2009;10(3):186–198. 19190637 10.1038/nrn2575

[38] Bassett DS, Sporns O. Network neuroscience. Nat Neurosci. 2017;20(3):353–364. 28230844 10.1038/nn.4502 PMC5485642

[39] Zhang H, Benz HL, Bezerianos A, et al Connectivity mapping of the human ECoG during a motor task with a time-varying dynamic Bayesian network. Annu Int Conf IEEE Eng Med Biol Soc. 2010;2010:130–133. 21096524 10.1109/IEMBS.2010.5627179 PMC4021587

[40] Milà BR, Sindhu KR, Mytinger JR, Shrey DW, Lopour BA. EEG biomarkers for the diagnosis and treatment of infantile spasms. Front Neurol. 2022;13:960454. 35968272 10.3389/fneur.2022.960454 PMC9366674

[41] Guimerà R, Mossa S, Turtschi A, Amaral LAN. The worldwide air transportation network: Anomalous centrality, community structure, and cities’ global roles. Proc Natl Acad Sci U S A. 2005;102(22):7794–7799. 15911778 10.1073/pnas.0407994102 PMC1142352

[42] Koschützki D, Schreiber F. Centrality analysis methods for biological networks and their application to gene regulatory networks. Gene Regul Syst Bio. 2008;2008(2):193–201. 10.4137/grsb.s702 PMC2733090 19787083

[43] Landherr A, Friedl B, Heidemann J. A critical review of centrality measures in social networks. Bus Inf Syst Eng. 2010;2(6):371–385.

[44] Engel J . Surgical treatment of the epilepsies. Raven Press; 1987.

[45] Li A, Huynh C, Fitzgerald Z, et al Neural fragility as an EEG marker of the seizure onset zone. Nat Neurosci. 2021;24(10):1465–1474. 34354282 10.1038/s41593-021-00901-w PMC8547387

[46] Kini LG, Bernabei JM, Mikhail F, et al Virtual resection predicts surgical outcome for drug-resistant epilepsy. Brain. 2019;142(12):3892–3905. 31599323 10.1093/brain/awz303 PMC6885672

[47] Ren S, Gliske SV, Brang D, Stacey WC. Redaction of false high frequency oscillations due to muscle artifact improves specificity to epileptic tissue. Clin Neurophysiol. 2019;130(6):976–985. 31003116 10.1016/j.clinph.2019.03.028 PMC6551620

[48] Scott JM, Gliske SV, Kuhlmann L, Stacey WC. Viability of preictal high-frequency oscillation rates as a biomarker for seizure prediction. Front Hum Neurosci. 2021;14:612899. 33584225 10.3389/fnhum.2020.612899 PMC7876341

[49] Gliske SV, Irwin ZT, Davis KA, Sahaya K, Chestek C, Stacey WC. Universal automated high frequency oscillation detector for real-time, long term EEG. Clin Neurophysiol. 2016;127(2):1057–1066. 26238856 10.1016/j.clinph.2015.07.016 PMC4723299

[50] Staba RJ, Wilson CL, Bragin A, Fried I. Quantitative analysis of high-frequency oscillations (80–500 Hz) recorded in human epileptic hippocampus and entorhinal cortex. J Neurophysiol. 2002;88(4):1743–1752. 12364503 10.1152/jn.2002.88.4.1743

[51] Bénar CG, Chauvière L, Bartolomei F, Wendling F. Pitfalls of high-pass filtering for detecting epileptic oscillations: A technical note on “false” ripples. Clin Neurophysiol. 2010;121(3):301–310. 19955019 10.1016/j.clinph.2009.10.019

[52] Bragin A, Mody I, Wilson CL, Engel J. Local generation of fast ripples in epileptic brain. J Neurosci. 2002;22(5):2012–2021. 11880532 10.1523/JNEUROSCI.22-05-02012.2002 PMC6758883

[53] Christodoulakis M, Hadjipapas A, Papathanasiou ES, Anastasiadou M, Papacostas SS, Mitsis GD. On the effect of volume conduction on graph theoretic measures of brain networks in epilepsy. Neuromethods. 2015;91:103–130.

[54] Anastasiadou MN, Christodoulakis M, Papathanasiou ES, Papacostas SS, Hadjipapas A, Mitsis GD. Graph theoretical characteristics of EEG-based functional brain networks in patients with epilepsy: The effect of reference choice and volume conduction. Front Neurosci. 2019;13:221. 30949021 10.3389/fnins.2019.00221 PMC6436604

[55] Giné E, Nickl R. Confidence bands in density estimation. Ann Stat. 2010;38(2):1122–1170.

[56] Chen YC . A tutorial on kernel density estimation and recent advances. Biostat Epidemiol. 2017;1(1):161–187.

[57] Valente TW, Coronges K, Lakon C, Costenbader E. How correlated are network centrality measures? Connect (Tor). 2008;28(1):16–26. 20505784 PMC2875682

[58] Freeman LC . Centrality in social networks conceptual clarification. Soc Networks. 1978;1(3):215–239.

[59] Newman M . Networks. 2nd edn. Oxford University Press; 2018.

[60] Bonacich P . Technique for analyzing overlapping memberships. Sociol Methodol. 1972;4:176–185.

[61] Davis KA, Devries SP, Krieger A, et al The effect of increased intracranial EEG sampling rates in clinical practice. Clin Neurophysiol. 2018;129(2):360–367. 29288992 10.1016/j.clinph.2017.10.039 PMC5955774

[62] Khambhati AN, Davis KA, Oommen BS, et al Dynamic network drivers of seizure generation, propagation and termination in human neocortical epilepsy. PLoS Comput Biol. 2015;11(12):e1004608. 26680762 10.1371/journal.pcbi.1004608 PMC4682976

[63] Hao S, Subramanian S, Jordan A, et al Computing network-based features from intracranial EEG time series data: Application to seizure focus localization. Annu Int Conf IEEE Eng Med Biol Soc. 2014;2014:5812–5815. 25571317 10.1109/EMBC.2014.6944949

[64] Mao JW, Ye XL, Li YH, Liang PJ, Xu JW, Zhang PM. Dynamic network connectivity analysis to identify epileptogenic zones based on stereo-electroencephalography. Front Comput Neurosci. 2016;10:113. 27833545 10.3389/fncom.2016.00113 PMC5081385

[65] Noe K, Sulc V, Wong-Kisiel L, et al Long-term outcomes after nonlesional extratemporal lobe epilepsy surgery. JAMA Neurol. 2013;70(8):1003–1008. 23732844 10.1001/jamaneurol.2013.209 PMC3920594

[66] Gliske SV, Irwin ZT, Chestek C, et al Variability in the location of high frequency oscillations during prolonged intracranial EEG recordings. Nat Commun. 2018;9(1):2155. 29858570 10.1038/s41467-018-04549-2 PMC5984620

[67] Engel J Jr, Bragin A, Staba R, Mody I. High-frequency oscillations: What is normal and what is not? Epilepsia. 2009;50(4):598–604. 19055491 10.1111/j.1528-1167.2008.01917.x

