# Supplementary material for: High frequency oscillation network dynamics predict outcome in non-palliative epilepsy surgery
Source: Brain Commun. 2024 Feb 7;6(1):fcae032. doi: 10.1093/braincomms/fcae032 (PMC10881100; doi:10.1093/braincomms/fcae032)
Supplement: fcae032_Supplementary_Data [file fcae032_supplementary_data.docx]

**Functional Connectivity Analysis**

**General Preprocessing**

We measured how an HFO interacts with the raw EEG signal on all other channels, without requiring the other channels to have a detected, coincident HFO. It is expected that this analysis will compare an HFO on one channel with data on other channels that may include a detected HFO, a subthreshold (undetected) HFO, a spike wave without HFO, or no measurable HFO at all. In other words, the only stipulation on this analysis is that there is an HFO on one channel for a given piece of data. It uses the HFO on one channel as an index to perform standard functional connectivity analysis across all channels.

This approach is based upon our observation that network discharges are not well represented by ‘detected’ HFOs. As seen in examples Supplementary Figure 1 and main Fig. 2, there are many channels participating in a high frequency discharge, but sometimes only a few of the channels have an HFO detected, and often it is only the middle portion of the discharge that is officially detected as an HFO. HFO detectors are designed to be specific, which removes many lower-amplitude discharges. However, for a network connectivity analysis, we must evaluate all activity on all channels. In addition, the initiation and phase of fast oscillations discharges is difficult to establish, especially when comparing across channels. As a result, we opted to use a detected HFO as a time index, then perform all analyses in the root-mean-squared form of the data. This assured that we were comparing the power of the signal and had removed all phase information from individual oscillations. This process enabled the first-ever analysis of network connectivity during HFOs.

Data for each patient was preprocessed independently (Supplementary Figure 1). In each patient, for each channel *i* of all *N* channels, we randomly selected 500 (if available) interictal HFO events on that channel. We used each of these events to create a time window of 100ms centered around the beginning of the detection. We then extracted the EEG data from *every channel* during this time window. For the rest of the article, we will refer to these *N*-channel by 100ms arrays of data as a ‘sample.’ Thus, each channel *i* has 500 samples, each of which has an HFO detection from channel *i*, but is agnostic to whether there are HFOs on any other channel.

Each row (channel) of the sample was then re-referenced using common average referencing with respect to each of their channel type (subdural grid or depth electrodes). Harmonic power grid noise was filtered using a 60 Hz IIR comb filter. Each row was then bandpass filtered using an 80-500 Hz elliptical passband filter.^1–3^ To further reduce noise and obtain the magnitude of the high frequency activity, we calculated the root mean squared (RMS) from the bandpass filtered data with 10ms sliding intervals.

It is critical to note that, although HFOs were detected using standard methods (bandpass filtered raw data with several steps as described in our previous work^1–3^), all functional connectivity analysis was performed using the RMS of that bandpass-filtered data. This assured the analysis was focused on power rather than phase of high frequency activity.

**Functional Connectivity Network (FCN)**

To calculate functional connectivity of the high frequency activity for each pair of channels (i,j), we focused on cross-correlations within our HFO-marked samples that were anomalously different from the background, i.e. different from cross-correlations during periods without HFOs (non-HFO background samples). In particular, to evaluate the connectivity of an HFO-specific channel i to any other channel j, we first computed the autocorrelation-normalized cross-correlation of the RMS data of i against j within a sample. From this we extracted the maximum amplitude of the cross correlation and its corresponding lag time, repeated for all 500 HFO samples: this produces a distribution of 500 lag times for each i,j pair. This distribution represents the temporal activity patterns of each channel j with respect to channel i. To account for zero-lag correlations that may just be volume conduction from electrical fields, we ignored all lags between -1 and 1ms as these effects are instantaneous.^4,5^

Having constructed the lag distribution of HFO-marked high frequency activity, we wanted to select distinctive samples out of the distribution that are the most characteristic of the interactions between channels i and j. To do this, we first had to distinguish them from the background connectivity between each channel when there were no HFOs. This is done by first taking 500 samples from periods with no HFO detections on every channel and processing them in the same fashion as with our HFO marked samples to generate a lag distribution of background correlations. Subsequently, for both the HFO lag distribution and the background lag distribution, we estimated their respective densities using kernel density estimation (KDE) and then estimated a 95% pointwise confidence band for the density via bootstrapping.^6,7^ At each lag value, we performed a two sample z-test ($\alpha$ = .05) between the HFO KDE and background KDE at each lag value to determine if the HFO KDE was significantly higher than that of the background KDE. This was evaluated at all lags to obtain significant lag intervals that are meant to reflect the most common functional connectivity paradigm(s) that are more specific to HFO based network activities. These HFO network specific samples corresponding to the lag intervals that were significantly different from the background are then used to compute the connectivity score.

The functional connectivity was calculated at each i,j pair by normalizing the mean of maximum cross correlation amplitudes *F_O_*(*ij*) of the specific samples from the previous paragraph. To normalize them, we randomly permuted the raw data on channel j and re-computed the cross correlation between i and j 1000 times. We calculated the mean *F_w_*(*ij*) and standard deviation *σ_w_*(*ij*) of these permuted peak correlation amplitudes. The standardized functional connectivity *F_Z_*(*ij*) is given by Eq. 1:

$F_{z}(ij)= \frac{F_{o}(ij)- F_{w}(ij)}{\sigma_{w}(ij)}$ (1)

Finally, because FCN is directionless, we repeated the whole process for electrode pair (j,i) and took the greater magnitude of the two (Eq. 2).

$FCN\left( ij \right)=FCN\left( ji \right)=max(|F_{Z}\left( ij \right)|,|F_{Z}\left( ji \right)|)$ (2)

By doing this for every pair of channels, we can construct a patient-specific functional connectivity network (FCN) where each cell represents the connectivity strength between a pair of channels. Additionally, we set the diagonal of the matrices to zero to ignore self-to-self connections.

**Lag Asymmetry Network (LAN)**

Given that epileptic seizures are characterized by wide propagating activities, we wanted to explore whether HFO activities can reveal underlying propagational networks. While the functional connectivity network captures the magnitude of the strengths of connectivity, it does not measure the directionality of such connections. Thus, we implemented a measurement of the magnitude of the lag directionality which we termed Lag Asymmetry and constructed a separate connectivity graph which became the Lag Asymmetry network (LAN). The pairwise channel asymmetry LAN_ij_ is calculated by Eq. 3:

$LAN_{ij}= \frac{N_{pos}-N_{neg}}{2\sqrt{\frac{N_{pos}}{N_{tot}}*\frac{N_{neg}}{N_{tot}}*N_{tot}}}$ (3)

The asymmetry measure is a standardized z-score in which the raw value is that of the difference between two perfectly correlated binomial variables. Here, the two variables, $N_{pos}$ and $N_{neg}$ are the count of positive and negative signs of the lag time of maximal cross correlation from the FCN analysis, and $N_{tot}$ is the total number of samples analyzed. As with the FCN analysis, we removed all cross correlations with maximum values at lags between -1 and 1ms to account for volume conduction. Additionally, we limited the calculations to samples with maximum cross correlation lags within 10ms as longer lags may be spurious and less reflective of underlying physiological connectivity. The LAN scores represent the significance of the leading or lagging relationship between a pair of channels i and j, with a large negative value indicating strong evidence of channel i preceding the activity of channel j while a large positive value signifies strong evidence of channel i following the activity of channel j.

**Centrality**

In network theory, a common category of tools used to evaluate the role of each node within a network is known as centrality.^8–10^ In our case, each electrode represents a node and the connection between two electrodes is known as an edge. We can assess different types of centralities for both the functional connectivity network (FCN) and the lag asymmetry network (LAN). First, to assess the importance of each node within FCN, we calculated the eigenvalue centrality of every node within the network. Channels with strong connections with many other channels are considered influential and having connections with more influential channels makes the channel even more important and therefore exhibits a higher eigenvector centrality.^9,11,12^ Therefore, these FCN eigenvector centralities (FCN-EIG) represent the most influential nodes within the network.

Eigenvector centrality is more commonly performed on undirected networks. Therefore, to perform a similar analysis for the LAN, we must first transform the LAN matrices into unsigned, undirected networks (uLAN$)$ by setting LAN_ij_ and LAN_ji_ to the greater magnitude of two values as we did with the functional connectivity network. Thereafter, we also calculated the eigenvector centrality of the nodes within this undirected uLAN network (uLAN-EIG).

Lastly, using the original LAN matrix we isolated all negative edges (i.e. i is leading j) to create a forward propagating network (fLAN). As a way to evaluate how well each node can act as a driver of propagating activity, we calculated each channel’s out-closeness centrality (fLAN-OUT).^9,10,12^ In a sense, out-closeness centrality is a way to measure how far upstream a node is from every other node within the network.

For all these different centrality types above, we standardized the raw scores by first performing an ordinal ranking of these values from 0 up to n-1 with the highest number representing the most important node and 0 being the least important given that all values are unique and then normalizing these ranked scores by the largest rank. Thus, each of n channels receives a unique score from 0 ( 0/(*n*-1), the lowest rank and least important) to 1 ( *n*-1/*n*-1, the highest rank and most important).

**Clinical prediction algorithm tutorial**

The following are tutorials and examples for how to generate the different measures.

**Examples of SOZ percentages (SOZALL, SOZ10, SOZ50, SOZTOP)**

Each HFO feature (HFO-RATE, FCN-EIG, uLAN-EIG, fLAN-OUT) is first calculated for every channel, and all channels ranked independently for each of the four features. To generate the SOZ percentages, determine all channels within the SOZ. For each HFO feature, find the rank of each channel that is within the SOZ. The SOZ percentage is the average normalized rank of highest *n_SOZ%_* channels within the SOZ, where *n_SOZ%_*= ceiling (*N*_SOZ channels_ * percentage).

Assume a patient has 100 channels and the clinicians have finished their evaluation, and five channels are determined to be in the SOZ. SOZ percentiles will evaluate those five channels in each of the four HFO features (HFO-RATE, FCN-EIG, uLAN-EIG, and fLAN-OUT), and check the results for four different percentiles (SOZALL, SOZ50, SOZ10, SOZTOP). Assume the channels in the SOZ have the following normalized ranks for FCN-EIG (0.1, 0.3, 0.43, 0.75, 0.85). With the rounding up, 50% of five channels is three channels while 10% and TOP are both the single highest channel. The SOZ percentile values are:

FCN-EIG-SOZALL 0.486 (average of 0.1, 0.3, 0.43, 0.75, 0.85)

FCN-EIG-SOZ50 0.8 (average of 0.75, 0.85)

FCN-EIG-SOZ10 0.85 (average of 0.85)

FCN-EIG-SOZTOP 0.85 (highest rank = 0.85)

Now assume a patient had 100 electrodes and 13 are in the SOZ. Within those channels, the normalized fLAN-OUT ranks are (0.1, 0.15, 0.23, 0.37, 0.4, 0.58, 0.79, 0.8, 0.85, 0.90, 0.91, 0.93, 0.99 ). With rounding up, for 13 electrodes, 50% is seven and 10% is two.

fLAN-OUT-SOZALL 0.6154 (average of 0.1, 0.15, 0.23, 0.37, 0.4, 0.58, 0.79, 0.8, 0.85, 0.90, 0.91, 0.93, 0.99)

fLAN-OUT-SOZ50 0.8814 (average of 0.79, 0.8, 0.85, 0.90, 0.91, 0.93, 0.99)

fLAN-OUT-SOZ10 0.96 (average of 0.93, 0.99)

fLAN-OUT-SOZTOP 0.99 (highest rank = 0.99)

Now assume a patient had 87 electrodes and three are in SOZ. Within those channels, the normalized fLAN-OUT ranks are (0, 0.79, 0.97).

fLAN-OUT-SOZALL 0.59 (average of 0, 0.79, 0.97)

fLAN-OUT-SOZ50 0.88 (average of 0.79, 0.97)

fLAN-OUT-SOZ10 0.97 (average of 0.97)

fLAN-OUT-SOZTOP 0.97 (highest rank = 0.97)

**Examples of Critical Resection Percentage (CReP)**

Assume a patient has 101 channels and the clinicians have finished their evaluation. They plan on resecting five channels. CReP will evaluate those five channels in each of the four measurements (HFO-RATE, FCN-EIG, uLAN-EIG, and fLAN-OUT), and check the results for four different percentiles (-CReP40, -CReP30, -CReP20, -CReP10). Assume the channels to be resected have the following normalized ranks for FCN-EIG (0.43, 0.75, 0.77, 0.85, 0.95). With 101 channels, the normalized scores go from 0 to 1, with increments of 1/(*n*-1) = 0.01 increments. The CReP values are:

FCN-EIG-CREP10 0.1 (1 of top 10: 0.95)

FCN-EIG-CREP20 0.2 (2 of top 20: 0.85, 0.95)

FCN-EIG-CREP30 0.13 (4 of top 30: 0.75, 0.77, 0.85, 0.95)

FCN-EIG-CREP40 0.1 (4 of top 40: 0.75, 0.77, 0.85, 0.95)

Now assume a patient had 71 electrodes and 13 are to be resected. Within those channels, the normalized fLAN-OUT ranks are (0.1, 0.15, 0.23, 0.37, 0.4, 0.58, 0.79, 0.8, 0.85, 0.90, 0.91, 0.93, 0.99). Assuming all ranks are unique, with rounding up, for 71 electrodes, 10% is eight channels and 50% is 36. The ranks have increments of 1/70 from 0 to 1, so (0, 0.014, 0.028, …. 0.971, 0.985, 1).

fLAN-OUT-CREP10 0.5 (4 of top 8: 0.90, 0.91, 0.93, 0.99)

fLAN-OUT-CREP20 0.4 (6 of top 15: 0.8, 0.85, 0.90, 0.91, 0.93, 0.99)

fLAN-OUT-CREP30 0.32 (7 of top 22: 0.79, 0.8, 0.85, 0.90, 0.91, 0.93, 0.99)

fLAN-OUT-CREP40 0.24 (7 of top 29: 0.79, 0.8, 0.85, 0.90, 0.91, 0.93, 0.99)

Now assume a patient had 87 electrodes and three are to be resected. Assuming all ranks are unique, within those channels, the normalized fLAN-OUT ranks are (0, 0.79, 0.97). Ranks are in increments of 1/86: (0, 0.012, 0.023, … 0.977, 0.988, 1)

fLAN-OUT-CREP10 0.11 (1 of top 9)

fLAN-OUT-CREP20 0.06 (1 of top 17)

fLAN-OUT-CREP30 0.07 (2 of top 27)

fLAN-OUT-CREP40 0.06 (2 of top 35)

| **Patient** | | **Age** | **Sex** | **ILAE class.** | **Engel outcome** | **Seizure focus (hemisphere, region)** | **Pathology** | **Number of intracranial channels** | | | | | **Percent SOZ resection** | **DS** | **Total recorded time (hours)** | **Total HFO count** | |  |
| --- | --- | --- | --- | --- | --- | --- | --- | --- | --- | --- | --- | --- | --- | --- | --- | --- | --- | --- |
|  |  |  |  |  |  |  |  |  |  |  |  |  |  |  |  |  |  |  |
|  |  |  |  |  |  |  |  | **Total** | **ECoG** | **Depth** | **SOZ** | **RV** |  |  |  |  |  |  |
| **UMHS-0018** | | 41 | M | 1 | Ib | L F | CD | 32 | 0 | 32 | 4 | 5 | 50% | N | 67 | 110,177 | |  |
| **UMHS-0019** | | 59 | F | 2 | II | R T | Gliosis | 106 | 106 | 0 | 2 | 40 | 100% | Y | 175.4 | 124,642 | |  |
| **UMHS-0020** | | 45 | F | 3 | II | R T | MTS | 25 | 0 | 25 | 9 | 9 | 100% | Y | 178.7 | 28,734 | |  |
| **UMHS-0022** | | 40 | M | 1 | I | L T | CD, MTS | 38 | 0 | 38 | 7 | 23 | 100% | Y | 167.4 | 63,700 | |  |
| **UMHS-0025** | | 17 | F | 2 | II | L T | Gliosis | 20 | 0 | 20 | 5 | 4 | 80% | Y | 214.9 | 178,705 | |  |
| **UMHS-0028** | | 14 | F | 1 | I | R T | Tumor: Glioma | 53 | 47 | 6 | 5 | 18 | 100% | Y | 88.8 | 216,041 | |  |
| **UMHS-0030** | | 5 | M | 5 | III | L T | MTS, Gliosis | 100 | 100 | 0 | 2 | 36 | 100% | Y | 152.5 | 441,168 | |  |
| **UMHS-0031** | | 13 | M | 1 | I | L T | Gliosis, Tumor: NF1 | 99 | 99 | 0 | 6 | 54 | 100% | Y | 191.8 | 569,313 | |  |
| **UMHS-0032** | | 41 | F | 1 | I | R F | CD | 32 | 0 | 32 | 3 | 16 | 100% | Y | 189.9 | 542,990 | |  |
| **UMHS-0033** | | 5 | F | 4 | II | R Insula | CD, Gliosis | 74 | 0 | 74 | 4 | 4 | 100% | Y | 125.3 | 86,855 | |  |
| **UMHS-0034** | | 33 | F | 5 | III | R F | Gliosis | 32 | 0 | 32 | 11 | 4 | 36% | N | 147.2 | 421,877 | |  |
| **UMHS-0035** | | 50 | F | 1 | I | L Hipp. | Gliosis | 57 | 57 | 0 | 2 | 11 | 100% | Y | 187.6 | 127,121 | |  |
| **UMHS-0037** | | 14 | M | 1 | I | L F | DNET | 50 | 0 | 50 | 7 | 14 | 29% | N | 235.3 | 227,536 | |  |
| **UMHS-0038** | | 28 | M | 2 | II | L T | MTS, Gliosis | 61 | 61 | 0 | 3 | 30 | 100% | Y | 189.0 | 215,510 | |  |
| **UMHS-0040** | | 14 | F | 1 | I | L P | CD, Gliosis | 63 | 55 | 8 | 8 | 14 | 87.5% | Y | 204.9 | 377,528 | |  |
| **UMHS-0041** | | 32 | F | 1 | I | R F | CD | 71 | 0 | 71 | 9 | 27 | 78% | N | 159.4 | 86,499 | |  |
| **UMHS-0042** | | 17 | M | 4 | II | L Insula |  | 60 | 0 | 60 | 8 | 7 | 37.5% | N | 73.9 | 31,465 | |  |
| **UMHS-0043** | | 28 | M | 1 | I | R T | Gliosis | 86 | 0 | 86 | 9 | 30 | 89% | Y | 168.2 | 493,238 | |  |
| **UMHS-0046** | | 23 | F | 1 | I | L F | CD | 30 | 0 | 30 | 9 | 12 | 100% | Y | 143.3 | 14,762 | |  |
| **UMHS-0047** | | 48 | F | 2 | II | R T | Gliosis | 70 | 0 | 70 | 3 | 30 | 100% | Y | 308.4 | 236,590 | |  |
| **UMHS-0050** | | 31 | F | 1 | I | L Hipp. | Gliosis | 93 | 71 | 22 | 3 | 8 | 0% | N | 169.1 | 287,992 | |  |
| **UMHS-0052** | | 27 | M | 1 | I | L Hipp. | MTS, Gliosis | 61 | 61 | 0 | 3 | 10 | 100% | Y | 151.3 | 164,878 | |  |
| **UMHS-0053** | | 55 | F | 1 | Id | R T | Gliosis | 70 | 0 | 70 | 3 | 29 | 100% | Y | 169.4 | 481,267 | |  |
| **UMHS-0054** | | 35 | F | 1 | I | L Hipp. | Gliosis | 61 | 61 | 0 | 9 | 14 | 100% | Y | 187.2 | 171,316 | |  |
| **UMHS-0055** | | 42 | M | 5 | III | L T, Hipp. | HS, Gliosis | 63 | 0 | 63 | 7 | 22 | 57% | N | 193.5 | 753,699 | |  |
| **UMHS-0060** | | 23 | M | 5 | III | R T | Tumor: RG, Gliosis | 80 | 80 | 0 | 9 | 32 | 56% | N | 174.5 | 1,090,979 | |  |
| **UMHS-0066** | | 43 | F | 5 | III | R F | FCD, Gliosis | 53 | 0 | 53 | 3 | 9 | 100% | Y | 217.7 | 1,011,536 | |  |
| **UMHS-0068** | | 23 | F | 6 | IV | R Hipp. |  | 102 | 0 | 102 | 4 | 5 | 100% | Y | 179.3 | 650,594 | |  |
| **UMHS-0071** | | 56 | F | 1 | I | L Hipp. | Gliosis | 89 | 0 | 89 | 2 | 14 | 0% | N | 288.2 | 290,862 | |  |
| **UMHS-0073** | | 39 | F | 5 | III | L Hipp. | Gliosis | 100 | 0 | 100 | 6 | 11 | 100% | Y | 256.4 | 194,040 | |  |
| **UMHS-0075** | | 24 | M | 1 | I | L T | Gliosis, MTS | 123 | 0 | 123 | 11 | 11 | 100% | Y | 179.9 | 802,164 | |  |
| **UMHS-0078** | | 35 | M | 1 | I | L Hipp. | MTS | 113 | 0 | 113 | 6 | 9 | 50% | N | 218.9 | 337,362 | |  |
| **UMHS-0079** | | 25 | F | 5 | III | L Hipp. |  | 189 | 0 | 189 | 11 | 5 | 36% | N | 186.9 | 887,655 | |  |
| **UMHS-0085** | | 21 | M | 5 | III | R F | FCD | 118 | 0 | 118 | 26 | 33 | 54% | N | 92.0 | 648,016 | |  |
| **UMHS-0093** | | 8 | M | 5 | III | R P | FCD | 111 | 0 | 111 | 3 | 5 | 100% | Y | 67.2 | 101,452 | |  |
|  | |  |  |  |  |  | **Totals** | 2585 | 798 | 1787 | 222 | 605 |  |  | 6100.4 | 12,468,273 | |  |
|  | |  |  |  |  |  | **Averages** |  |  |  |  |  |  |  | 174.3 | 356,236.1 | |  |
|  |  | *CD: cortical dysplasia, DNET: dysembryoplastic neuroepithelial tumor, DS: Definitive Surgery, F: frontal, FCD: Frontal cortical dysplasia, Hipp.: Hippocampus, HS: hippocampal sclerosis, L/R: left / right, M/F: male, female, MTS: medial temporal sclerosis, NF1: neurofibromatosis type 1 tumor, P: parietal, PMG: polymicrogyria, PVNH: periventricular nodular heterotopia, RG: recurrent ganglioglioma, RV: Resected volume (# electrodes within), SOZ: Seizure onset zone, T: temporal* | | | | | | | | | | | | | | |  |  |
|  |  |  |  |  |  |  |  |  |  |  |  |  |  |  |  |  |  |  |

**Supplementary Table 1. Full patient demographics and clinical data.**


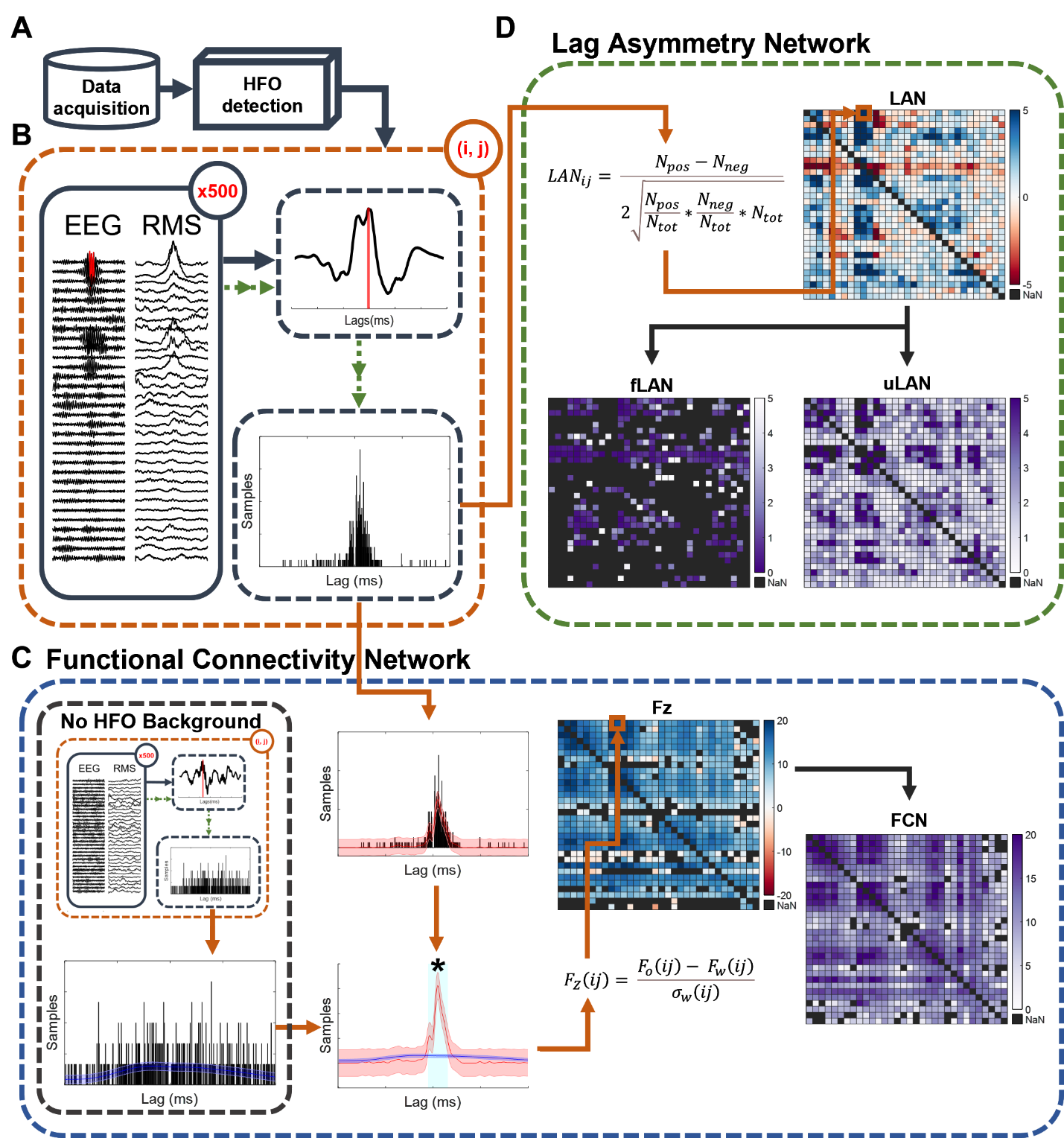


**Supplementary Figure 1. Diagram of HFO network characterization workflow.** (A) Data are acquired and automated HFO detection is performed. (B) For each channel i, 500 HFOs that were detected on i are randomly selected. Data from all channels that occurred +/- 100 ms around each detection are used to create 500 ‘samples’ for connectivity analysis. Samples are generated by bandpass filtering the raw data (80-500 Hz) and calculating the RMS of every channel, then performing the cross correlation between each pair of channels. This generates 500 samples from each of the N channels, each with N cross-correlations, normalized by autocorrelation. The distribution of the 500 lag times at maximum cross-correlation for each i,j pair was used for the next steps. (C) To obtain the functional connectivity score F_z_ between channel i and j, we only evaluated cross-correlations that stood out from the background, i.e. only for the data that generated the light blue box in the bottom middle plot (*). Background connectivity was evaluated by using the same process described in B but using 500 samples during which no HFO was detected. For both distributions, we computed their probability densities with kernel density estimation along with confidence bands (red bands: HFO distribution, dark blue bands: background distribution). At each lag, z-test was performed between the two distributions to identify which lag intervals from the HFO distribution were significantly higher than the background distribution. Samples generating these lags were then used to calculate F_z_ for the given i,j pair. For F_z_, we calculate F_o_ (the mean of all cross correlation amplitudes from samples with significant lags) and normalize against the mean and standard deviation (F_w_ and $\sigma$_w_) of the maximal cross correlations of the permuted signals at the same lag. The final values FCN_ij_ and FCN_ji_ are evaluated as the larger magnitude of F_z_(ij) and F_z_(ji). (D) Using Eq. 3, the lag asymmetry network is made by counting the number of positive lags (N_pos_) and negative lags (N_neg_) between -10 and 10ms to compute the LAN score between channels i and j (LAN_ij_) . We subsequently derive forward propagating network fLAN by only evaluating the inverse of the magnitude of the negative values. The symmetric network uLAN is derived in the same fashion as FCN in (C), such that uLAN­_ij_ and uLAN_ji_ take on the largest magnitude value of LAN_ij_ and LAN_ji_.


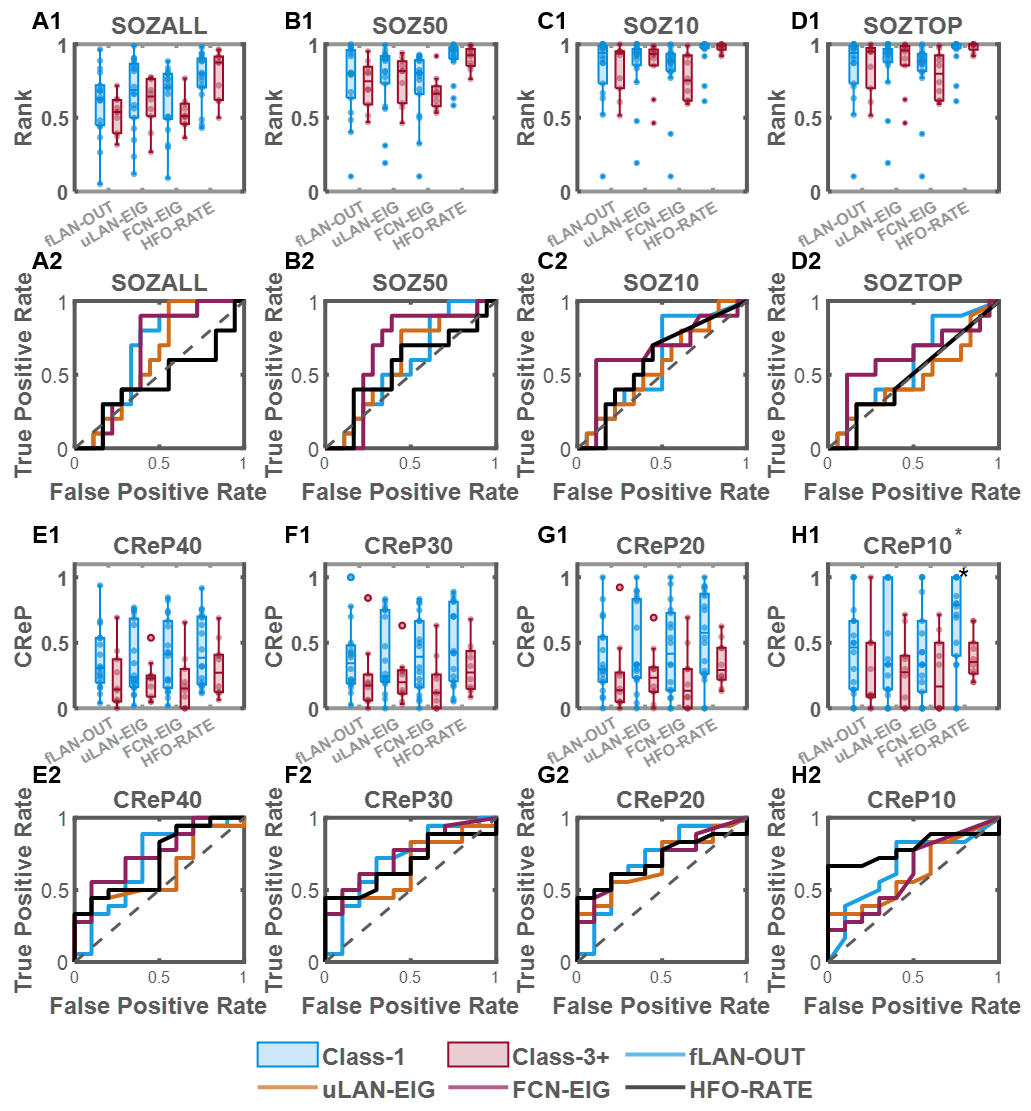


**Supplementary Figure 2.** **SOZ and CReP features and their respective ROC curves for all of the patients comparing between class 1 and class 3+ patients.** Boxes represent the interquartile range with the horizontal line being the median and whiskers extending 1.5 times the interquartile range. Raw data points are overlaid on top of the box plots. Hotelling’s T2 reveals no significant group effect (*p > 0.05, n = 28)* for all sets except CReP10 (*p = 0.293, T2 = 10.767, n = 28).* Significant differences were denoted by * for *p< 0.05*, ** for *p< 0.01*, and *** for *p< 0.001*.


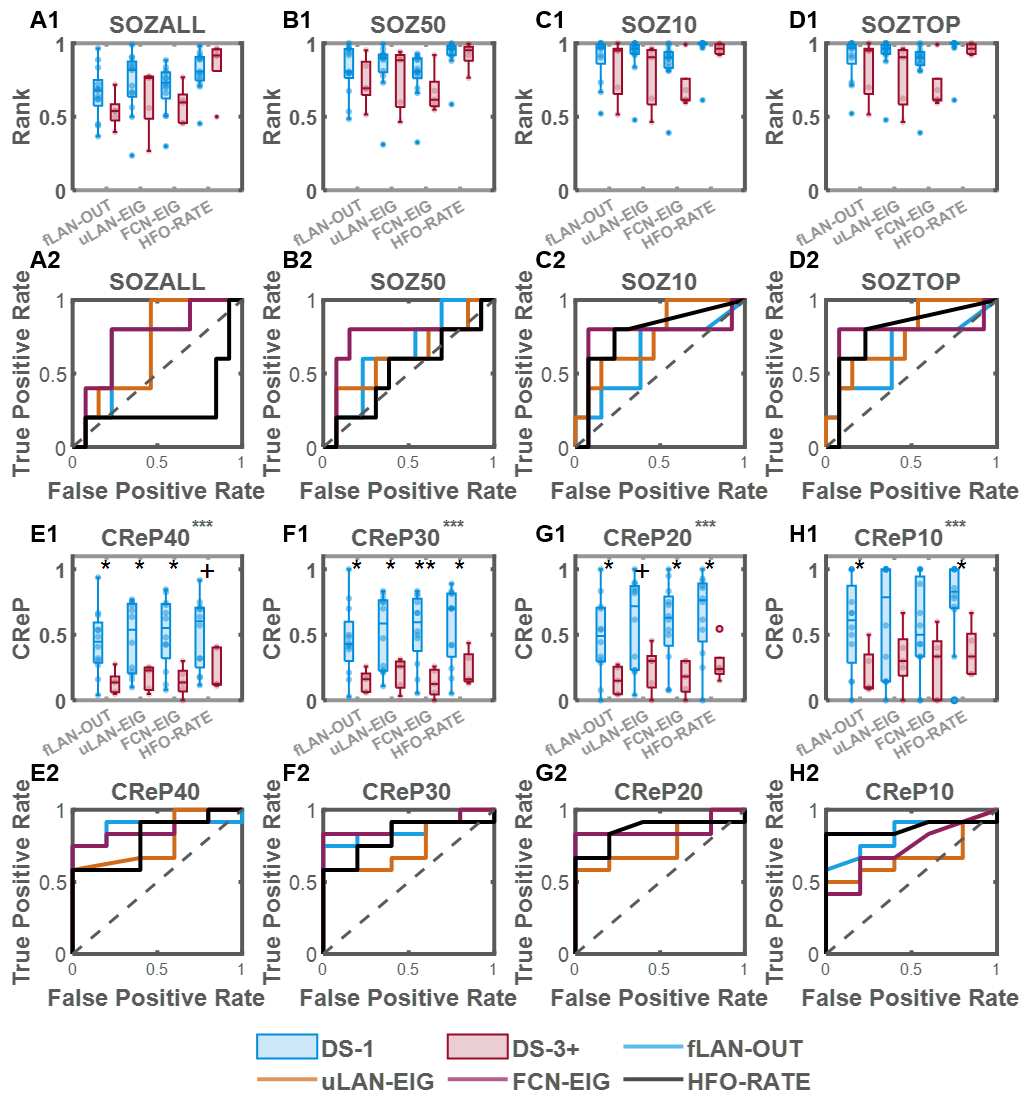


**Supplementary Figure 3.** **SOZ and CReP features and their respective ROC curves for the definitive surgery (DS) patients only comparing between DS-1 and DS-3+.** Boxes represent the interquartile range with the horizontal line being the median and whiskers extending 1.5 times the interquartile range. Raw data points are overlaid on top of the box plots. No significant group differences were detected for the SOZ features (*p > 0.05, n = 17 Hotelling’s T2).* However, all CReP sets showed significant group differences (*p = 0.001, 1.25*10^-5^, 1.17*10^-5^, 0.0005, T2 = 18.630, 27.996, 28.131, 20.016, n = 17 for CReP40,30,20,10 respectively*). Significant differences were denoted by * for *p< 0.05*, ** for *p< 0.01*, and *** for *p< 0.001*.


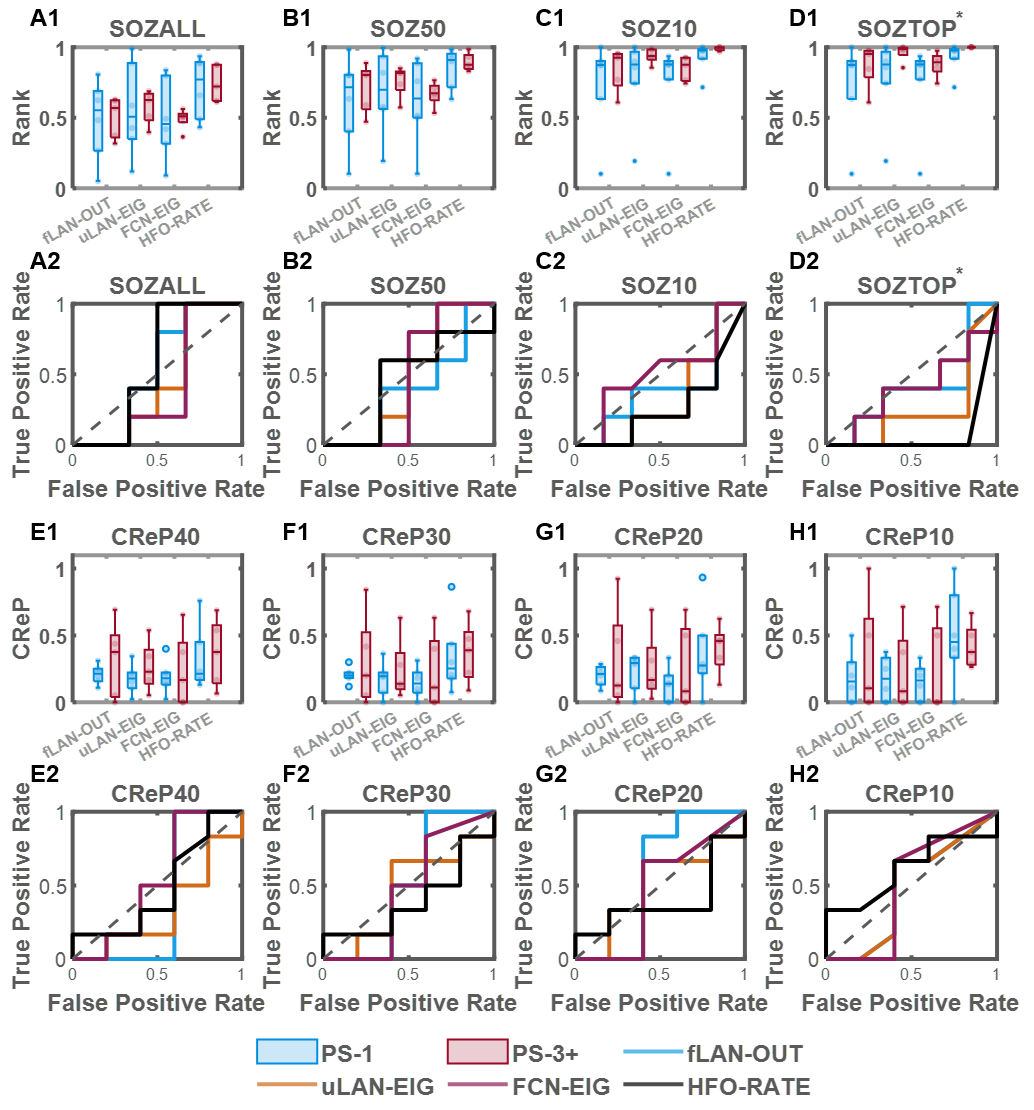


**Supplementary Figure 4.** **SOZ and CReP features and their respective ROC curves for the palliative surgery (PS) patients only comparing between PS-1 and PS-3+.** Boxes represent the interquartile range with the horizontal line being the median and whiskers extending 1.5 times the interquartile range. Raw data points are overlaid on top of the box plots. Hotelling’s T2 reveals no significant group difference (*p > 0.05, n = 11)* for all groups except SOZTOP (D2) (*p = 0.029, T2 = 10.793, n = 11).* Significant differences were denoted by * for *p< 0.05*, ** for *p< 0.01*, and *** for *p< 0.001*.


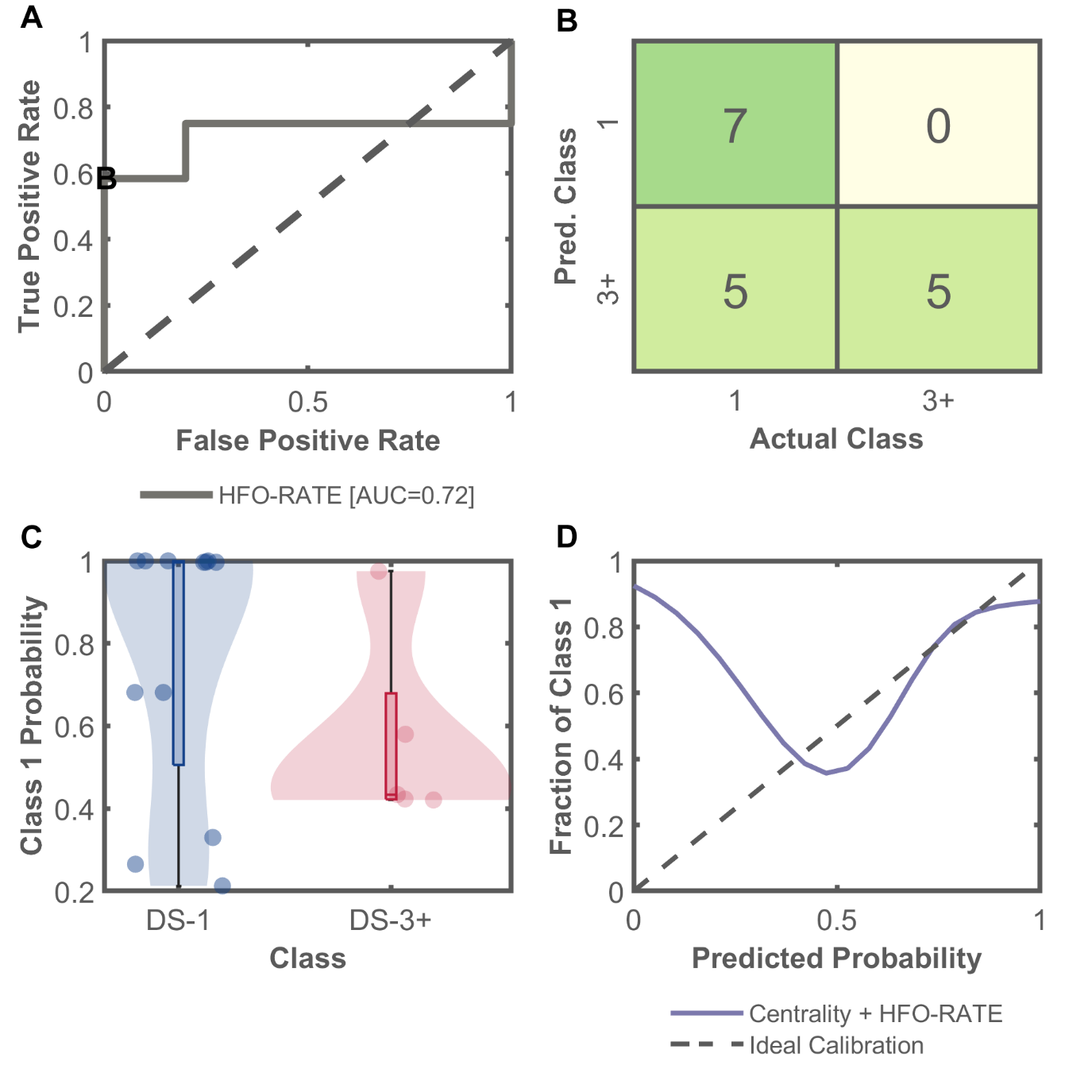


**Supplementary Figure 5. Naïve Bayes model results using HFO-RATE only for definitive surgery (DS) patients.** (A) ROC curve of leave-one-out naïve Bayes posterior probabilities. (B) Confusion matrix at the selected point from (A). (C) Distributions of the posterior probabilities for class 1 and class 3+ definitive surgery (DS-1, DS-3+) respectively. (D) Calibration curve of posterior probabilities. Note there are fewer patients with high probability compared with main Fig. 6 (which incorporated centralities and HFO-RATE), and one of them is a false positive.


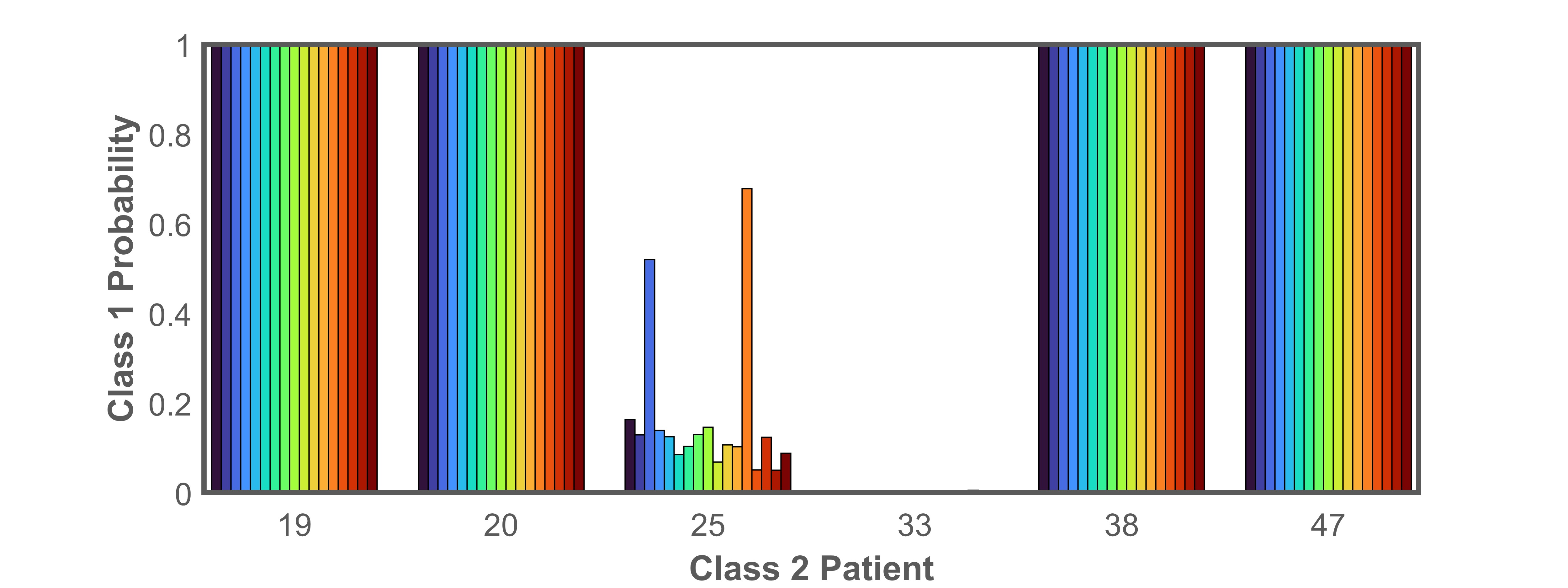


**Supplementary Figure 6. Leave-one-out Naïve Bayes outputs for held out definitive surgery (DS) Class 2 patients.** The DS-2 patients were not used in the 17-fold leave-out cross validation. Thus there are 17 different models that can be tested against the DS-2 patients as a held-out data set. This figure displays the results of all 17 models. Four patients (19, 20, 38 and 47) received 100% Class 1 probability predictions for all validation models. Patients 25 and 33 had minor variability but were classified as poor outcome.

**A**

**B**

**Supplementary Figure 7. Class 2 definitive surgery (DS) patient critical resection percentage (CReP30).** (A) Comparison between Class 1 (*blue, n = 12*) and Class 2 (*yellow,* n *= 6*) patient CReP30 of centrality and HFO rate ranks. Hoteling’s T2 showed no significant group difference (*p = 0.568, T2 = 2.938, n = 18)*. (B) Comparison between Class 2 and Class 3+ (*red, n = 5*) patient CReP30 of centrality and HFO rate ranks with significant group difference detected (*p = 0.018*, T2 = 11.906, *n = 11 Hotelling’s T2*). Significant differences were denoted by * for *p< 0.05*, ** for *p< 0.01*, and *** for *p< 0.001*.

**Supplementary References**

1. Gliske S v, Irwin ZT, Chestek C, et al. Variability in the location of high frequency oscillations during prolonged intracranial EEG recordings. *Nat Commun*. 2018;9(1):2155. doi:10.1038/s41467-018-04549-2

2. Gliske S v., Irwin ZT, Davis KA, Sahaya K, Chestek C, Stacey WC. Universal automated high frequency oscillation detector for real-time, long term EEG. *Clinical Neurophysiology*. 2016;127(2):1057-1066. doi:10.1016/j.clinph.2015.07.016

3. Ren S, Gliske S v., Brang D, Stacey WC. Redaction of false high frequency oscillations due to muscle artifact improves specificity to epileptic tissue. *Clinical Neurophysiology*. 2019;130(6):976-985. doi:10.1016/j.clinph.2019.03.028

4. Christodoulakis M, Hadjipapas A, Papathanasiou ES, Anastasiadou M, Papacostas SS, Mitsis GD. On the effect of volume conduction on graph theoretic measures of brain networks in epilepsy. *Neuromethods*. 2015;91:103-130. doi:10.1007/7657_2013_65

5. Anastasiadou MN, Christodoulakis M, Papathanasiou ES, Papacostas SS, Hadjipapas A, Mitsis GD. Graph Theoretical Characteristics of EEG-Based Functional Brain Networks in Patients With Epilepsy: The Effect of Reference Choice and Volume Conduction. *Front Neurosci*. 2019;13(221). doi:10.3389/fnins.2019.00221

6. Giné E, Nickl R. Confidence bands in density estimation. *Ann Stat*. 2010;38(2):1122-1170. doi:10.1214/09-AOS738

7. Chen YC. A tutorial on kernel density estimation and recent advances. *Biostat Epidemiol*. 2017;1(1). doi:10.1080/24709360.2017.1396742

8. Newman M. *Networks (2nd Edition)*. Oxford University Press, Oxford; 2018.

9. Valente TW, Coronges K, Lakon C, Costenbader E. How Correlated Are Network Centrality Measures? *Connect (Tor)*. 2008;28(1):16-26.

10. Freeman LC. Centrality in social networks conceptual clarification. *Soc Networks*. 1978;1(3):215-239. doi:10.1016/0378-8733(78)90021-7

11. Bonacich P. Technique for Analyzing Overlapping Memberships. *Sociol Methodol*. 1972;4:176-185. doi:10.2307/270732

12. Landherr A, Friedl B, Heidemann J. A Critical Review of Centrality Measures in Social Networks. *Business & Information Systems Engineering*. 2010;2(6):371-385. doi:10.1007/s12599-010-0127-3
